# Prophylactic Mechanical Closure for Preventing Delayed Bleeding after Gastric Endoscopic Submucosal Dissection: A Systematic Review and Meta‐Analysis

**DOI:** 10.1002/deo2.70299

**Published:** 2026-02-14

**Authors:** Hariruk Yodying, Vichit Viriyaroj, Thammanij Rookkachart, Thana Boonsinsukh, Suun Sathornviriyapong, Anuwat Chartkitcharoen, Wannakorn Prapasajchavet, Natchanok Mekrugsakit, Patcharaon Petchkaewkul, Ratchanon Laojanun

**Affiliations:** ^1^ Department of Surgery HRH Princess Maha Chakri Sirindhorn Medical Center Faculty of Medicine Srinakharinwirot University Nakhon Nayok Thailand

**Keywords:** delayed bleeding, endoscopic submucosal dissection, gastric neoplasms, mechanical closure, meta‐analysis

## Abstract

**Background:**

Delayed bleeding remains the most common adverse event after gastric endoscopic submucosal dissection (ESD). We assessed whether prophylactic mechanical closure is associated with reduced clinically significant delayed bleeding.

**Methods:**

PubMed, Embase, Cochrane CENTRAL, Scopus, and ClinicalTrials.gov were searched through October 2025. Comparative studies reporting delayed bleeding within 30 days were included. Random‐effects meta‐analysis with Hartung‐Knapp‐Sidik‐Jonkman adjustment was applied. Evidence certainty was assessed using GRADE.

**Results:**

Nine studies (2646 patients; eight non‐randomized) met inclusion criteria. Prophylactic closure was associated with reduced delayed bleeding (risk ratio [RR] 0.36, 95% confidence interval [CI] 0.16–0.82; *p* = 0.02; *I* ^2^ = 65%; prediction interval 0.05–2.65). However, sensitivity analysis restricted to high‐quality designs (one RCT, two propensity‐matched studies) showed wide confidence intervals without statistical significance (RR 0.30, 95% CI 0.01–10.44; *p* = 0.28). Subgroup analyses revealed no significant effect modification by antithrombotic status (*p* = 0.96) or defect size (*p* = 0.27). In exploratory subgroup analysis, advanced techniques were associated with RR 0.14 (95% CI 0.03–0.55) versus standard approaches RR 0.62 (95% CI 0.21–1.77; interaction *p* = 0.005), though this difference was confounded by operator expertise. Immediate complete closure rate was 93.6% (95% CI 45.0%–99.6%). GRADE certainty was low due to the predominance of observational studies and substantial heterogeneity.

**Conclusions:**

Prophylactic closure was associated with reduced delayed bleeding after gastric ESD; however, low‐certainty evidence limits definitive conclusions. Well‐designed randomized trials in high‐risk populations are warranted to inform clinical practice.

**Trial Registration:**

PROSPERO registration: CRD420251172925

## Graphical abstract

Nine comparative studies (*n* = 2646) were included. Prophylactic mechanical closure was associated with lower clinically significant delayed bleeding after gastric endoscopic submucosal dissection (risk ratio 0.36, 95% confidence interval 0.16–0.82; *I* ^2^ = 65%). Evidence certainty was low; exploratory analyses suggested larger effects with advanced techniques than with standard approaches.

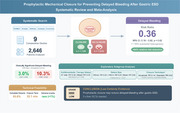

## 1 Introduction

Gastric endoscopic submucosal dissection (ESD) has become the standard of care for early gastric cancer and dysplastic lesions meeting endoscopic resection criteria, offering organ preservation with oncologic outcomes equivalent to surgery for appropriately selected patients [1, 2]. Despite technical advances and established efficacy, delayed bleeding remains the most common serious complication, occurring in 4%–6% of unselected patients and escalating to 15%–45% in high‐risk populations, particularly those requiring antithrombotic therapy [3, 4, 5]. This complication carries substantial clinical consequences, including emergency intervention, transfusion requirements, prolonged hospitalization, and rarely death [6, 7].

Current bleeding prevention strategies focus primarily on meticulous intraprocedural hemostasis of visible vessels [8] and pharmacologic acid suppression with proton pump inhibitors [9]. While these measures reduce bleeding risk, substantial residual event rates persist, particularly among high‐risk populations. Current antithrombotic management guidelines recommend continuation of most agents during gastric ESD when hemostatic measures are adequate [10, 11]. The BEST‐J bleeding prediction score identifies patients at elevated risk based on antithrombotic regimen, comorbidities, and lesion characteristics, with scores exceeding 3 points correlating with bleeding rates approaching 20%–40% [12]. For such patients, effective preventive strategies beyond conventional hemostasis represent an unmet clinical need.

Prophylactic mechanical closure of post‐ESD mucosal defects has emerged as a potential preventive intervention. The biological rationale is intuitive: approximating wound edges should reduce exposed submucosal vessels, accelerate healing, and decrease bleeding risk. However, early studies using standard through‐the‐scope clips showed inconsistent results, likely reflecting technical limitations in achieving complete closure of large defects [8, 13]. Recent development of advanced closure techniques—including the reopenable clip‐over‐line method (ROLM), endoscopic ligation with O‐ring closure (E‐LOC), and endoscopic hand suturing (EHS)—has reinvigorated interest in prophylactic closure, with several studies demonstrating complete closure rates exceeding 95% and substantial bleeding reductions [14, 15, 16].

Current international guidelines regarding prophylactic closure remain equivocal. The European Society of Gastrointestinal Endoscopy (ESGE) 2022 guideline suggests against routine prophylactic closure while acknowledging potential benefit in selected high‐risk patients [17]. The American Society for Gastrointestinal Endoscopy 2023 guideline addresses the ESD technique but does not provide specific closure recommendations [18]. Earlier AGA guidance similarly focused on ESD technique and training without specific closure recommendations [19]. Japanese guidelines suggest individualized decision‐making without strong endorsement [20, 21]. This guideline equipoise reflects both limited comparative evidence and heterogeneity of closure techniques evaluated.

This systematic review and meta‐analysis aimed to evaluate whether prophylactic mechanical closure reduces clinically significant delayed bleeding after gastric ESD across diverse patient populations, with pre‐specified subgroup analyses examining patient risk stratification by antithrombotic therapy status and defect characteristics, technique‐specific efficacy, and technical feasibility outcomes informing real‐world implementation decisions.

## 2 Methods

This systematic review and meta‐analysis were conducted following the Cochrane Handbook methodology and reported according to PRISMA 2020 guidelines [22] and the Meta‐analysis Of Observational Studies in Epidemiology reporting standards. The protocol was prospectively registered with PROSPERO (CRD420251172925) before study selection commenced.

### 2.1 Search Strategy and Information Sources

We systematically searched PubMed/MEDLINE, Embase, Cochrane CENTRAL, Scopus, and ClinicalTrials.gov through October 2025 without language restrictions. Searches were conducted on October 1, 2025 (PubMed/MEDLINE and Scopus) and updated on October 22, 2025 (Embase, Cochrane CENTRAL, and ClinicalTrials.gov). Reference lists were hand‐searched. Complete search strategies are provided in Appendix S1.

### 2.2 Eligibility Criteria

Eligible studies included randomized controlled trials and comparative observational cohort studies (including concurrent or historical controls) evaluating prophylactic mechanical closure after gastric ESD versus no closure. Because historical controls may introduce temporal confounding, we prespecified a sensitivity analysis excluding historical‐control studies. Studies were excluded if closure was performed therapeutically or if they assessed non‐mechanical closure methods.

The primary outcome was clinically significant delayed bleeding, defined as hematemesis, melena, or hemoglobin decrease ≥2 g/dL occurring after completion of the ESD procedure and requiring emergency endoscopy within 30 days. Secondary outcomes included delayed perforation, hospital length of stay, closure procedure time, closure‐related adverse events, immediate complete closure rate, and sustained closure at second‐look endoscopy.

### 2.3 Study Selection and Data Extraction

Two reviewers independently screened titles and abstracts, with full‐text review of potentially eligible studies. Disagreements were resolved by discussion. Data extraction was performed independently using standardized forms. For studies reporting medians and ranges, we estimated means and standard deviations using validated methods [23, 24].

### 2.4 Risk of Bias Assessment

Risk of bias was assessed using the Cochrane Risk of Bias 2 (RoB 2) tool for randomized trials [25] and the Risk Of Bias In Non‐randomized Studies of Interventions (ROBINS‐I) tool for observational studies [26]. Two reviewers independently assessed each study across all domains, with disagreements resolved by discussion. Publication bias was assessed visually using funnel plots. Egger's regression test was planned when ≥10 studies were available; because only nine studies contributed to the primary analysis, Egger's test was performed as an exploratory analysis and interpreted cautiously due to limited power.

### 2.5 Technique Classification

Closure techniques were prespecified and categorized into three groups: (1) Advanced/specialized techniques (e.g., ROLM, E‐LOC, EHS, alternate‐layer clip, and related line‐assisted/suturing methods) (2), standard/basic techniques (e.g., conventional through‐the‐scope clips and endoloop‐based closures without line‐assisted traction), and (3) variable/mixed techniques when multiple approaches were used without protocolized assignment. Studies classified as variable/mixed were analyzed separately in technique subgroup analyses to minimize misclassification bias. This classification was pre‐specified in the protocol and applied by two independent reviewers, with disagreements resolved by discussion.

### 2.6 Statistical Analysis

We performed random‐effects meta‐analysis using the Mantel‐Haenszel method for dichotomous outcomes, calculating pooled risk ratios (RRs) with 95% confidence intervals. We applied the Hartung‐Knapp‐Sidik‐Jonkman (HKSJ) adjustment as recommended for meta‐analyses with fewer than 10 studies [27]. Continuous outcomes were analyzed using mean differences with inverse‐variance weighting. Heterogeneity was assessed using Cochran *Q* and *I* ^2^ statistics [28]. Prediction intervals were calculated.

Pre‐specified subgroup analyses examined effect modification by antithrombotic therapy status, defect size (≥40 mm cutoff), closure technique sophistication, and study design quality. Between‐subgroup differences were tested using chi‐square tests. These subgroup analyses should be interpreted as exploratory and hypothesis‐generating rather than confirmatory, given the observational nature of most included studies and limited sample sizes within subgroups. Sensitivity analyses tested key assumptions by restricting to high‐quality designs, excluding historical controls, applying stricter outcome definitions, and leave‐one‐out analysis (Figures S1–S5).

Technical feasibility outcomes (closure rates, procedure time, and adverse events) were analyzed using single‐arm proportion meta‐analyses with generalized linear mixed models. Expanded analyses incorporated additional single‐arm studies with subgroup analyses by technique category. Publication bias was assessed using funnel plots and Egger's test [29]. Evidence certainty was assessed using GRADE [30, 31].

All analyses were performed using Review Manager version 5.4 (Cochrane Collaboration) and R version 4.3.1 with the meta and metafor packages. Statistical significance was defined as two‐sided *p* < 0.05.

## 3 Results

### 3.1 Study Selection and Characteristics

**FIGURE 1:**
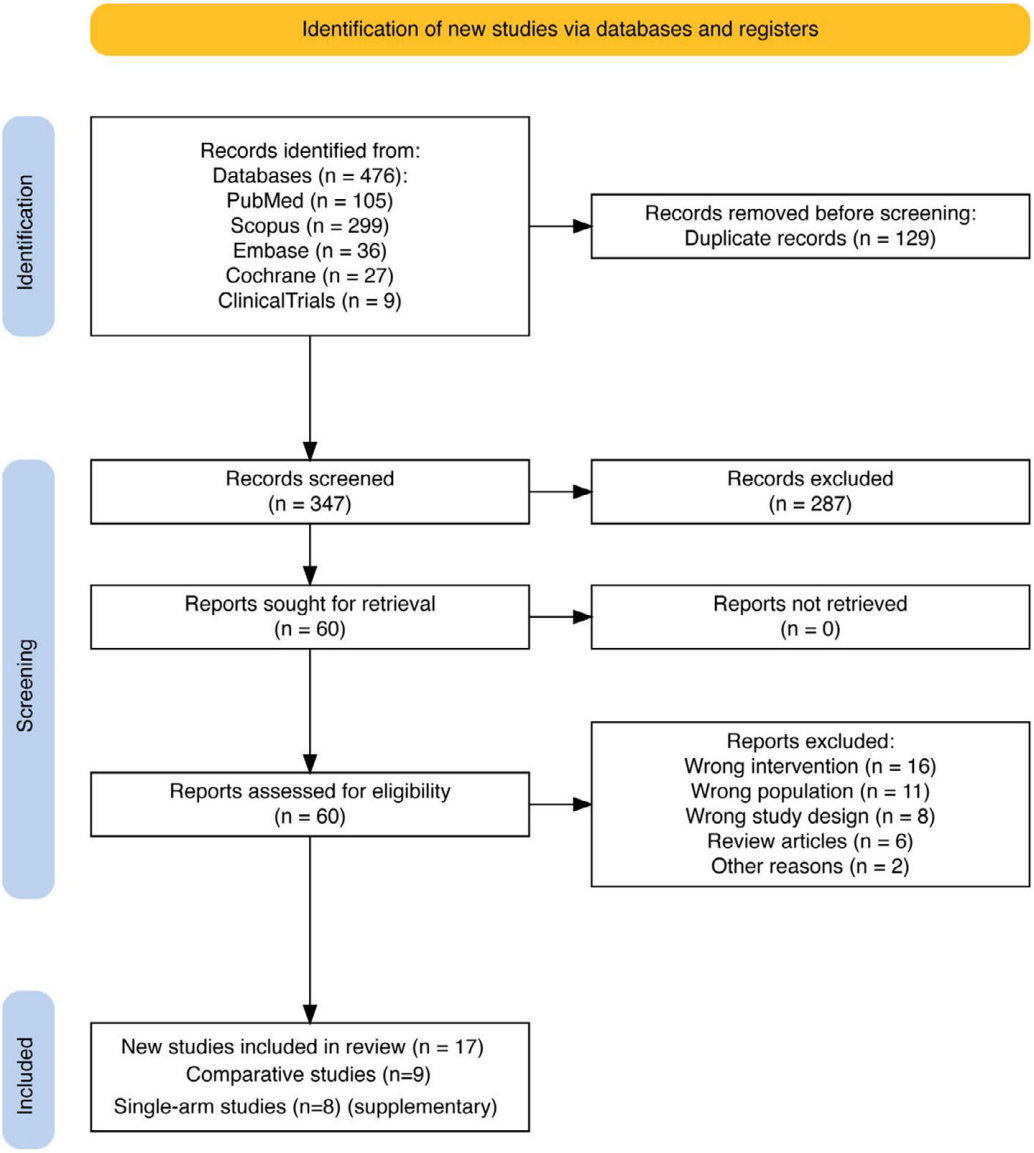
PRISMA flow diagram of study selection systematic search across five databases (PubMed, Embase, Cochrane CENTRAL, Scopus, ClinicalTrials.gov) identified 476 citations. After deduplication and screening, nine comparative studies (2646 patients) met the inclusion criteria for primary efficacy analysis. Eight additional single‐arm studies (Table S1) were included for expanded technical feasibility synthesis (total 17 studies).

Database searches identified 476 citations, reduced to 347 after deduplication (Figure 1). Title and abstract screening excluded 287 citations, leaving 60 for full‐text review. After full‐text assessment, 43 articles were excluded. Ultimately, nine comparative studies (2646 patients) met all inclusion criteria for the primary efficacy analysis [16, 32, 33, 34, 35, 36, 37, 38, 39]. An additional eight single‐arm studies (Table S1) were identified for the expanded technical feasibility synthesis [15, 40, 41, 42, 43, 44, 45, 46], providing comprehensive data on closure success rates, durability, procedure time, and adverse events across 17 total studies.

Characteristics of the nine included comparative studies are presented in Table 1. Notably, eight of nine included studies (89%) were non‐randomized observational designs, substantially limiting the ability to draw causal conclusions about the efficacy of prophylactic closure. Six were retrospective cohort studies [16, 32, 33, 36, 38, 39], two were prospective cohort studies with propensity score matching [34, 35], and one was a randomized controlled trial [37]. Studies were published between 2011 and 2025, with six originating from Japan, one from Korea, one from China, and one from the United States. Sample sizes ranged from 52 to 924 patients. The median age of participants was 66–74 years across studies, with male predominance (60%–75%). Four studies enrolled exclusively or predominantly patients on antithrombotic therapy, reflecting this population's elevated bleeding risk and clinical importance [16, 35, 36, 39].

**TABLE 1:** Characteristics of included comparative studies.

Study (Year)	Country	Design	*N* (Closure/Control)	Closure technique	AT status	Defect size (mm)^a^	Dominant third	CSDB events (*n*/*N*)^b^	SLE performed	Closure time (min)	Immediate complete closure	LOS (day)^c^
Chen 2025	China	Retrospective, multicenter	479/445	Advanced (alternate mucosa‐submucosa clips)	Non‐AT	≤40 (median 38)	Lower	4/479 versus 34/445	NR	NR	479/479^d^	8.33 ± 2.22 versus 8.67 ± 2.96
Sugimoto 2025	Japan	PSM, ulticenter	168/168	Advanced (ROLM)	Mixed	≥40 (mean 45.9)	NR	3/168 versus 13/168	No	35.6 ± 17.5	168/168	NR
Kobayashi 2023	Japan	PSM, single‐center	32/32	Mixed (clips ± E‐LOC)	AT‐only	≤40 (mean ∼32)	Lower	0/32 versus 5/32	NR	24 [14–36]^e^	44/44^d^	NR
Ego 2021	Japan	Retrospective, single‐center	131/269	Standard (endoloop+clips)	AT‐only	≤40 (mean 34.8)	Lower	15/131 versus 32/269	Yes (POD 3)	15 [4–60]	113/131	NR
Shiotsuki 2021	Japan	Retrospective, single‐center	37/141	Standard (ELC)	AT‐only	≤40 (mean 34.8)	Lower	3/37 versus 32/141	Yes	14 [8–47]	27/37	NR
Lee 2011	Korea	RCT	26/26	Standard (snare+clips)	Non‐AT	Mixed	Lower	1/26 versus 0/26	NR	14.4 (7.4–32.1)	16/26	3.00 ± 0.50 versus 6.00 ± 2.50
Wang 2023	China	Retrospective, single‐center	162/297	Standard (clips)	Unreported	Mixed	Lower	5/162 versus 24/297	NR	NR	NR	NR
Nishiyama 2022	Japan	Historical control	48/50	Advanced (E‐LOC)	AT‐only	≤40 (mean 31.6)	Lower	0/48 versus 10/50	Yes (POD 2–3)	29.9 ± 12.5	47/48	NR
Ramai 2025	USA	Retrospective, single‐center	45/90	Mixed (various)	Mixed	Mixed	Lower	3/45 versus 6/90	NR	NR	NR	NR

Abbreviations: AT, antithrombotic therapy; CSDB, clinically significant delayed bleeding; ELC, endoloop closure; E‐LOC, endoscopic ligation with O‐ring closure; LOS, length of stay; NR, not reported; POD, postoperative day; PSM, propensity score matching; RCT, randomized controlled trial; ROLM, reopenable clip‐over‐the‐line method; SLE, second‐look endoscopy.

^a^Defect size based on resected specimen diameter in closure arm.

^b^CSDB, clinically significant delayed bleeding within 30 days.

^c^LOS converted from median using Wan/Hozo methods where applicable.

^d^By‐definition 100% (closure group defined by successful closure).

^e^Median (IQR).

Closure techniques were classified prospectively per protocol. Three studies employed advanced specialized techniques (Sugimoto 2025: ROLM; Nishiyama 2022: E‐LOC; Chen 2025: alternate‐layer clips). Four studies used standard approaches (Wang 2023: through‐the‐scope clips; Lee 2011, Ego 2021, Shiotsuki 2021: endoloop‐based methods). Two studies (Kobayashi 2023, Ramai 2025) used mixed techniques without standardized protocols and were analyzed separately to avoid misclassification bias. Mean resected specimen size ranged from 32 to 46 mm across studies. Antithrombotic therapy was continued perioperatively in most studies, consistent with contemporary guidelines.

### 3.2 RoB Assessment

Using RoB 2 for the randomized trial and ROBINS‐I for observational studies, we classified one study at low risk [37], two at moderate risk [34, 35], and six at serious risk due to confounding by indication [16, 32, 33, 36, 38, 39] (Table S2). Risk of bias assessment revealed that six of nine studies (67%) were judged to have serious overall risk of bias, primarily due to confounding by indication and selection bias inherent to non‐randomized designs. This high proportion of studies with serious methodological limitations fundamentally constrains confidence in the pooled effect estimate. Three studies performed multivariable adjustment for key confounders [33, 36, 38].

### 3.3 Primary Outcome: Clinically Significant Delayed Bleeding

**FIGURE 2:**
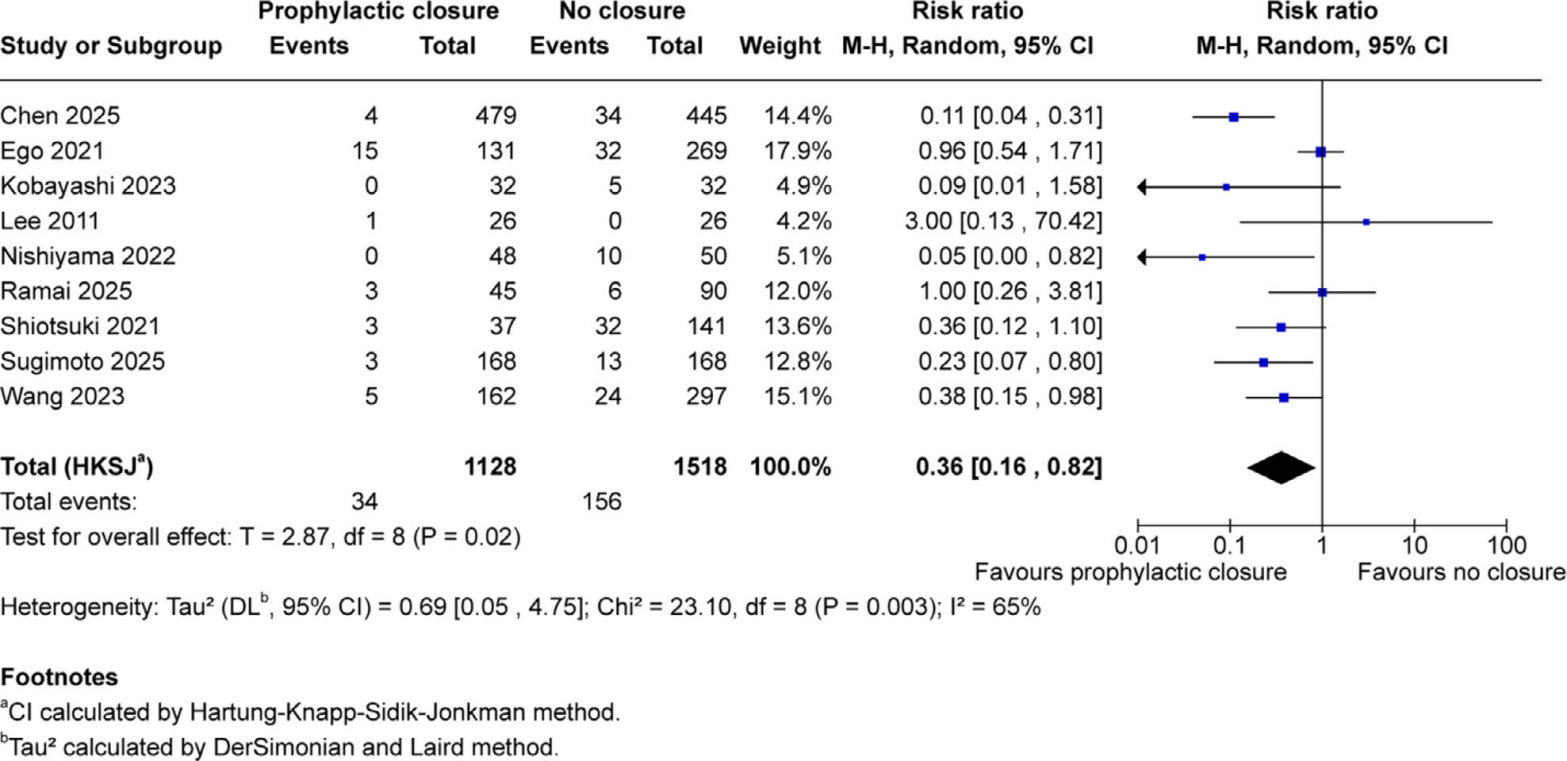
Forest plot of clinically significant delayed bleeding random‐effects meta‐analysis with Hartung‐Knapp‐Sidik‐Jonkman adjustment of nine comparative studies evaluating prophylactic mechanical closure versus no closure after gastric endoscopic submucosal dissection. Outcome: clinically significant delayed bleeding within 30 days. Total 2646 patients (1128 closure, 1518 control); 34 events versus 156 events. Pooled risk ratio 0.36 (95% CI 0.16–0.82), *T* = 2.87, df = 8, *p* = 0.02. Heterogeneity: *τ* ^2^ = 0.69 (95% CI 0.05–4.75), χ^2^ = 23.10, df = 8, *p* = 0.003, *I* ^2^ = 65%. ^a^CI calculated by Hartung‐Knapp‐Sidik‐Jonkman method. ^b^ *τ* ^2^ calculated by the DerSimonian and Laird method.

Nine comparative studies (2646 patients; 1128 closure and 1518 no closure) contributed data for the primary efficacy outcome (Figure 2). Clinically significant delayed bleeding occurred in 34 patients (3.0%) in the closure group compared to 156 patients (10.3%) in the no‐closure group. Random‐effects meta‐analysis with HKSJ adjustment demonstrated that prophylactic closure was associated with reduced delayed bleeding risk (pooled RR 0.36, 95% CI 0.16–0.82; *p* = 0.02), corresponding to a 64% relative risk reduction and absolute risk difference of 7.3%. Substantial heterogeneity was evident (*I* ^2^ = 65%, *τ* ^2^ = 0.69, 95% CI 0.05–4.75), and all individual study point estimates favored closure.

### 3.4 Subgroup Analysis by Antithrombotic Therapy Status

**FIGURE 3:**
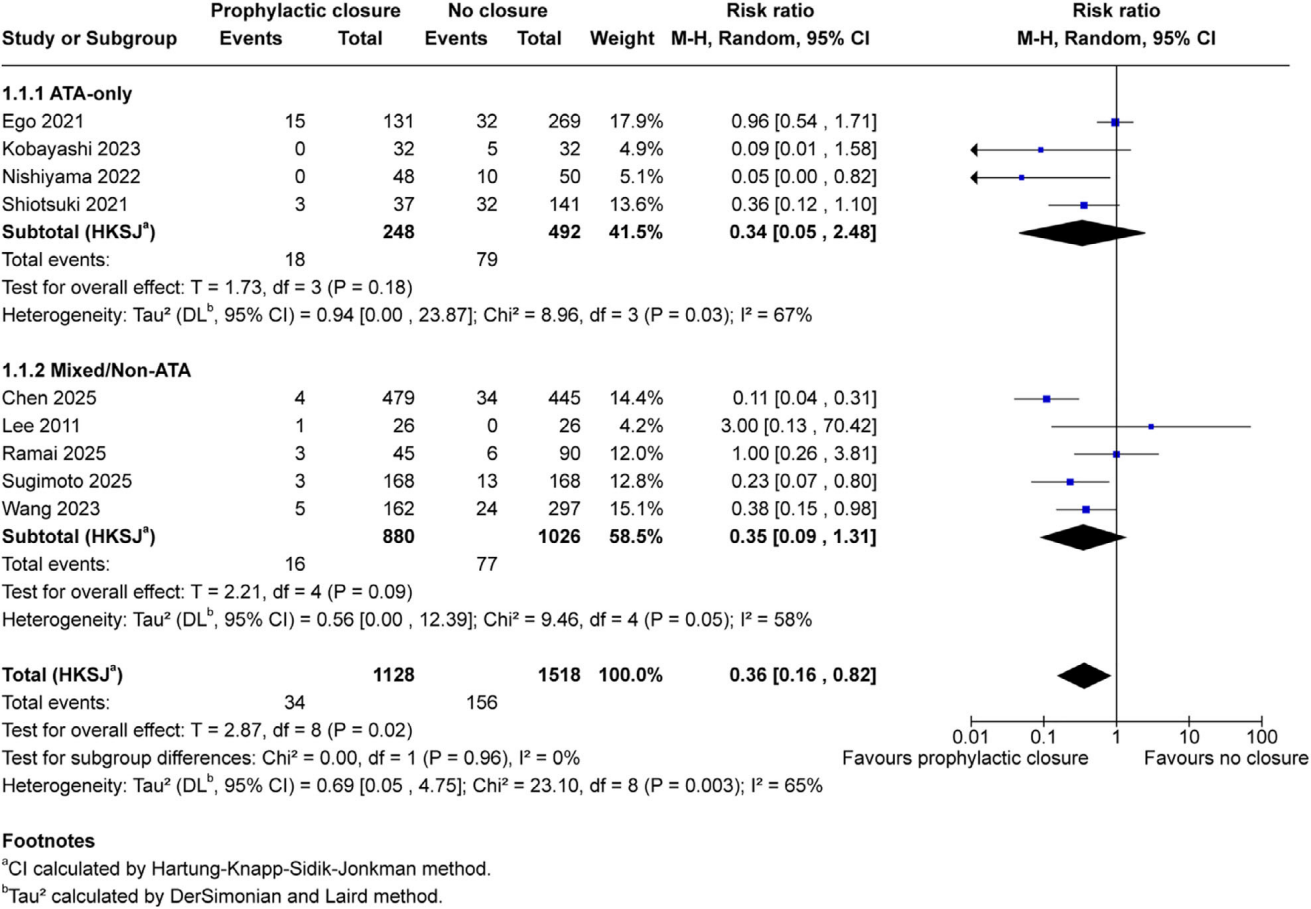
Subgroup analysis by antithrombotic therapy status: Exploratory subgroup analysis stratified by antithrombotic therapy status. ATA‐only cohorts (four studies, 740 patients: 248 closure, 492 control): RR 0.34 (95% CI 0.05–2.48), *T* = 1.73, df = 3, *p* = 0.18, *I* ^2^ = 67%. Mixed/Non‐ATA populations (five studies, 1906 patients: 880 closure, 1026 control): RR 0.35 (95% CI 0.09–1.31), *T* = 2.21, df = 4, *p* = 0.09, *I* ^2^ = 58%. Test for subgroup differences: χ^2^ = 0.00, df = 1, *p* = 0.96, *I* ^2^ = 0%. This analysis is exploratory; wide confidence intervals reflect limited power to detect effect modification. ^a^CI calculated by Hartung‐Knapp‐Sidik‐Jonkman method. ^b^ *τ* ^2^ calculated by the DerSimonian and Laird method.

Among four studies enrolling exclusively antithrombotic patients (740 patients), prophylactic closure demonstrated directionally consistent effects (RR 0.34, 95% CI 0.05–2.48; *p* = 0.18; *I* ^2^ = 67%) [16, 35, 36, 39]. (Figure 3). In mixed populations (five studies, 1906 patients), closure showed a similar association (RR 0.35, 95% CI 0.09–1.31; *p* = 0.09; *I* ^2^ = 58%) [32, 33, 34, 37, 38]. The test for subgroup differences was not significant (*p* = 0.96). This exploratory analysis suggests no statistically significant effect modification by antithrombotic status, though confidence intervals were wide and analyses were underpowered to detect clinically important interactions.

### 3.5 Subgroup Analysis by Defect Size

**FIGURE 4:**
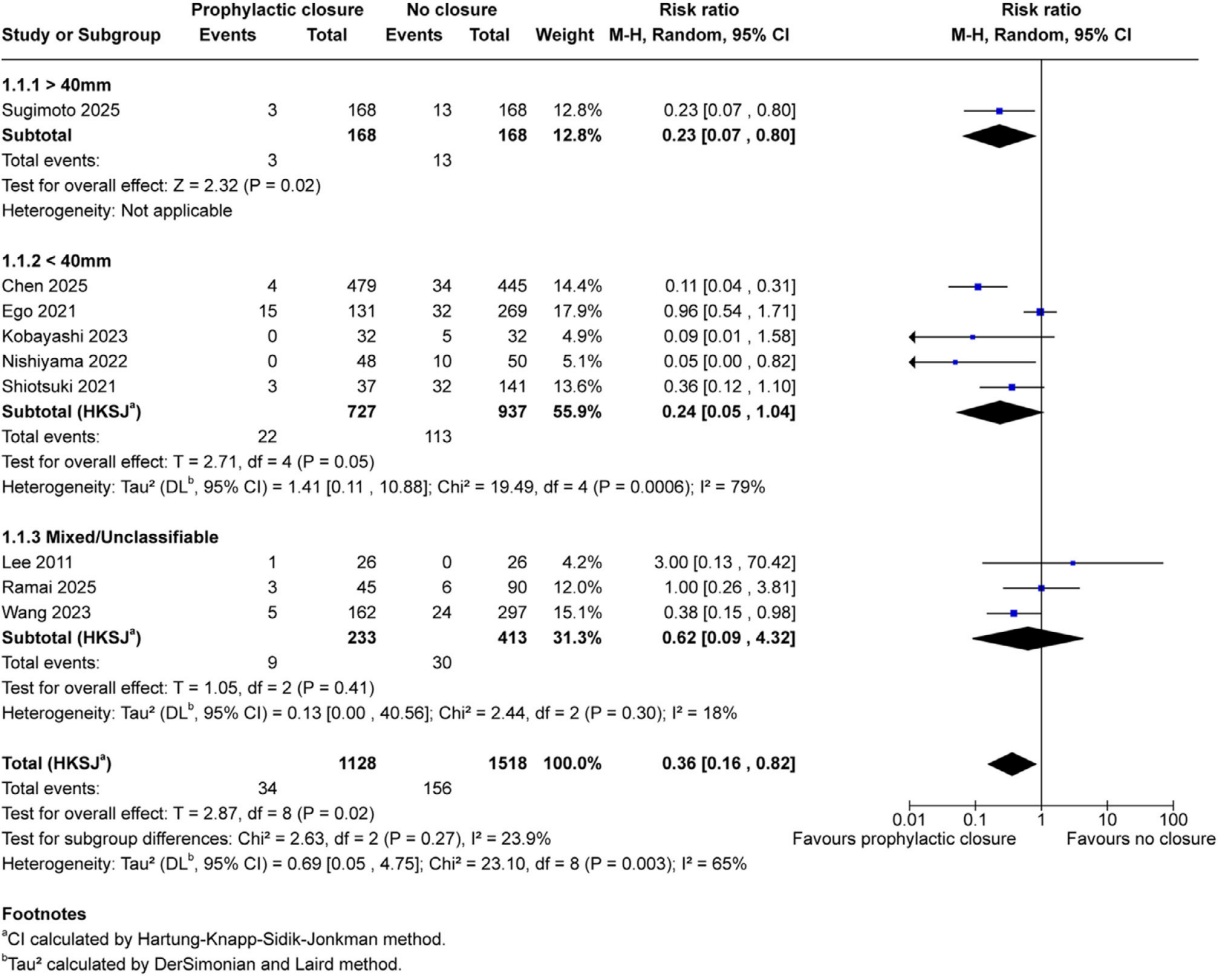
Subgroup analysis by defect size: Exploratory subgroup analysis stratified by resected specimen size. Defects >40 mm (one study, 336 patients: 168 closure, 168 control): RR 0.23 (95% CI 0.07–0.80), *Z* = 2.32, *p* = 0.02. Defects <40 mm (five studies, 1,664 patients: 727 closure, 937 control): RR 0.24 (95% CI 0.05–1.04), *T* = 2.71, df = 4, *p* = 0.05, *I* ^2^ = 79%. Mixed/Unclassifiable (three studies, 646 patients: 233 closure, 413 control): RR 0.62 (95% CI 0.09–4.32), *T* = 1.05, df = 2, *p* = 0.41, *I* ^2^ = 18%. Test for subgroup differences: χ^2^ = 2.63, df = 2, *p* = 0.27, *I* ^2^ = 23.9%. This analysis is exploratory and hypothesis‐generating. ^a^CI calculated by Hartung‐Knapp‐Sidik‐Jonkman method. ^b^ *τ* ^2^ calculated by the DerSimonian and Laird method.

One study with large defects >40 mm (Sugimoto 2025; 336 patients) showed marked bleeding reduction (RR 0.23, 95% CI 0.07–0.80; *p* = 0.02) [34]. (Figure 4). Studies with defects <40 mm (five studies, 1664 patients) showed favorable estimates (RR 0.24, 95% CI 0.05–1.04; *p* = 0.05; *I* ^2^ = 79%) [16, 33, 35, 36, 39]. Three studies with alternative size reporting (646 patients) showed RR 0.62 (95% CI 0.09–4.32; *p* = 0.41; *I* ^2^ = 18%) [32, 37, 38]. The test for subgroup differences was not significant (*p* = 0.27). These exploratory analyses did not detect statistically significant heterogeneity across defect size categories, though limited sample sizes preclude definitive conclusions about effect consistency.

### 3.6 Subgroup Analysis by Closure Technique

**FIGURE 5:**
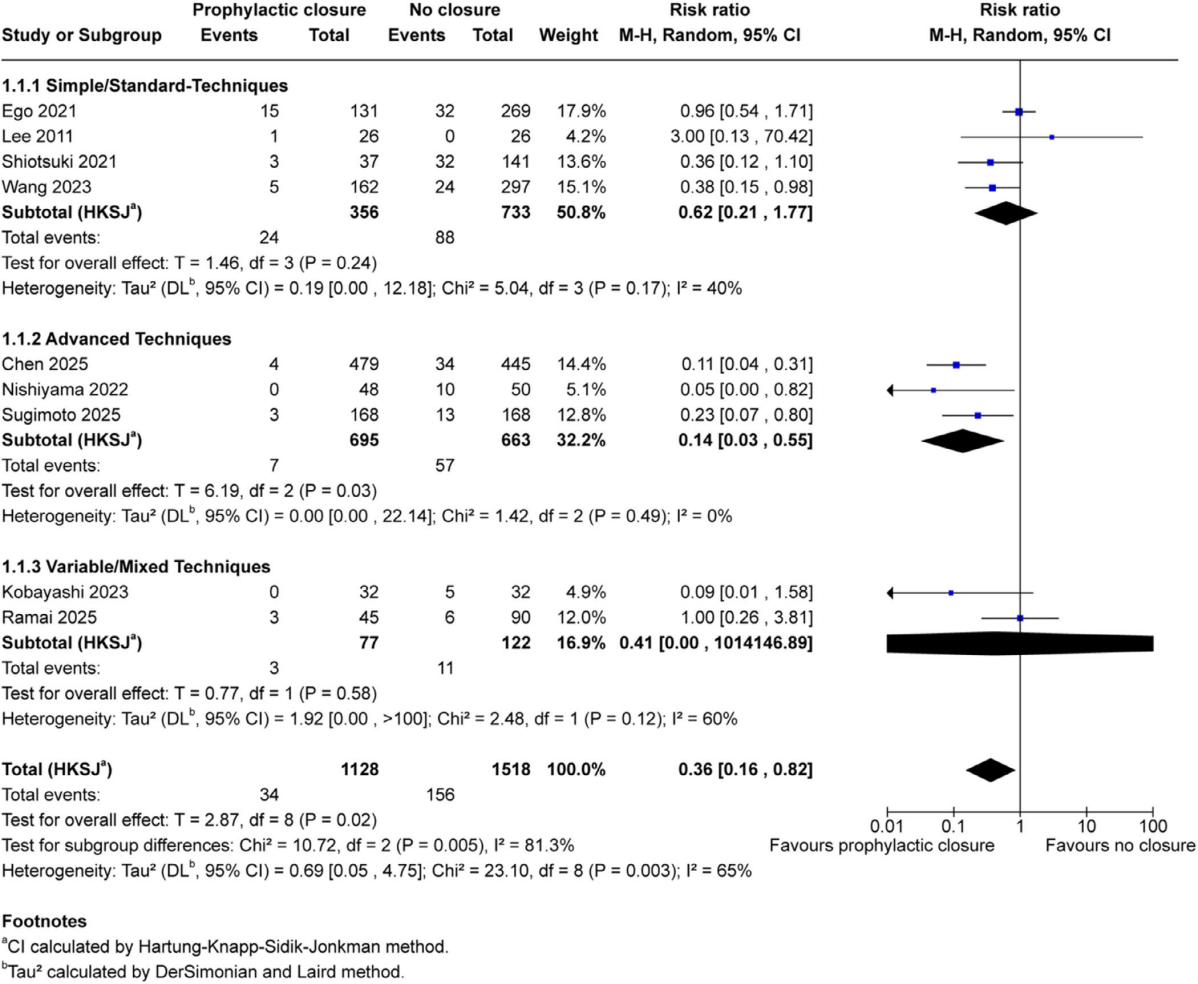
Subgroup analysis by closure technique: Exploratory subgroup analysis stratified by closure technique sophistication with Hartung‐Knapp‐Sidik‐Jonkman adjustment. Simple/Standard‐Techniques (four studies, 1089 patients: 356 closure, 733 control): RR 0.62 (95% CI 0.21–1.77), *T* = 1.38, df = 3, *p* = 0.24, *I* ^2^ = 40%. Advanced Techniques (three studies, 1358 patients: 695 closure, 663 control): RR 0.14 (95% CI 0.03–0.55), *T* = 6.19, df = 2, *p* = 0.03, *I* ^2^ = 0%. Variable/Mixed Techniques (two studies, 199 patients: 77 closure, 122 control): RR 0.41 (95% CI 0.00–1014146.89), *T* = 0.77, df = 1, *p* = 0.58, I^2^ = 60% (interpret descriptively only due to df = 1). Test for subgroup differences: χ^2^ = 10.72, df = 2, *p* = 0.005, *I* ^2^ = 81.3%. Caution: technique effects are confounded by operator expertise and center volume. ^a^CI calculated by Hartung‐Knapp‐Sidik‐Jonkman method. ^b^ *τ* ^2^ calculated by the DerSimonian and Laird method.

Closure technique sophistication appeared to modify treatment effects (Figure 5). Advanced techniques (ROLM, E‐LOC, and alternate‐layer clips; three studies, 1358 patients) demonstrated superior efficacy (RR 0.14, 95% CI 0.03–0.55; *p* = 0.03; *I* ^2^ = 0%) [16, 33, 34]. Standard techniques (clips, endoloop‐based methods; four studies, 1089 patients) showed more modest effects (RR 0.62, 95% CI 0.21–1.77; *p* = 0.24; *I* ^2^ = 40%) [36, 37, 38, 39]. The test for subgroup differences was significant (*p* = 0.005). Two studies with mixed protocols (199 patients) showed extreme imprecision precluding quantitative interpretation [32, 35]. This exploratory finding must be interpreted with substantial caution, as discussed below in the context of confounding by expertise and center volume.

### 3.7 Sensitivity Analyses

The primary finding remained directionally consistent across sensitivity analyses (Table 2 and Figures S1–S5). Restricting to high‐quality designs (RCT plus PSM studies; 452 patients) yielded consistent direction (RR 0.30, 95% CI 0.01–10.44; *p* = 0.28; I^2^ = 30%) [34, 35, 37]. This sensitivity analysis is critically important: when restricted to designs least susceptible to confounding, the evidence for efficacy does not reach conventional significance thresholds, though inadequate statistical power with only three studies provides an alternative explanation. Excluding historical control (Nishiyama 2022) maintained significant benefit (RR 0.40, 95% CI 0.18–0.91; *p* = 0.03; *I* ^2^ = 65%) [32, 33, 34, 35, 36, 37, 38, 39]. Excluding non‐protocolized study (Ramai 2025) strengthened the estimate (RR 0.31, 95% CI 0.13–0.76; *p* = 0.02; *I* ^2^ = 68%) [16, 33, 34, 35, 36, 37, 38, 39]. Leave‐one‐out analysis yielded stable results (RR range 0.31–0.48).

**TABLE 2:** Sensitivity analyses for primary outcome (clinically significant delayed bleeding).

Sensitivity analysis	Studies included (*n*)	Pooled RR (95% CI)	*I* ^2^ (%)	*p*‐Value	Key finding
Primary analysis (HKSJ)	All nine studies	0.36 (0.16–0.82)	65	0.02	Significant reduction in bleeding
SA‐1: High‐quality only	RCT + PSM (3)	0.30 (0.01–10.44)	30	0.28	Direction consistent, very wide CI
SA‐2: Exclude historical control	Eight studies	0.40 (0.18–0.91)	65	0.03	Effect maintained, significant
SA‐3: Strict CSDB definition	Eight studies	0.35 (0.13–0.95)	70	0.04	Effect maintained, significant
SA‐4: Exclude non‐protocolized	Eight studies	0.31 (0.13–0.76)	68	0.02	Effect strengthened, significant
SA‐5: AT‐only cohorts	Four studies	0.34 (0.05–2.48)	67	0.18	Consistent direction in high‐risk

Abbreviations: AT, antithrombotic therapy; CI, confidence interval; CSDB, clinically significant delayed bleeding; HKSJ, Hartung‐Knapp‐Sidik‐Jonkman; PSM, propensity score matching; RCT, randomized controlled trial; RR, risk ratio; SA, sensitivity analysis.

### 3.8 Secondary Outcomes

#### 3.8.1 Delayed Perforation

Three comparative studies reported delayed perforation explicitly—Chen 2025 (0/479 vs. 0/445), Kobayashi 2023 (0/32 vs. 0/32), and Ramai 2025 (0/45 vs. 0/90)—with no events in any arm [32, 33, 35]. These data suggest that delayed perforation after gastric ESD is very rare under contemporary practice in both closure and non‐closure groups.

#### 3.8.2 Length of Hospital Stay

Only two studies reported this outcome with contradictory results (Chen 2025: MD −0.34 days; Lee 2011: MD −3.0 days) and extreme heterogeneity (I^2^ = 96%), precluding pooled analysis (Figure S6) [33, 37].

### 3.9 Technical Feasibility Outcomes (Comparative Studies)

#### 3.9.1 Immediate Complete Closure

**FIGURE 6:**
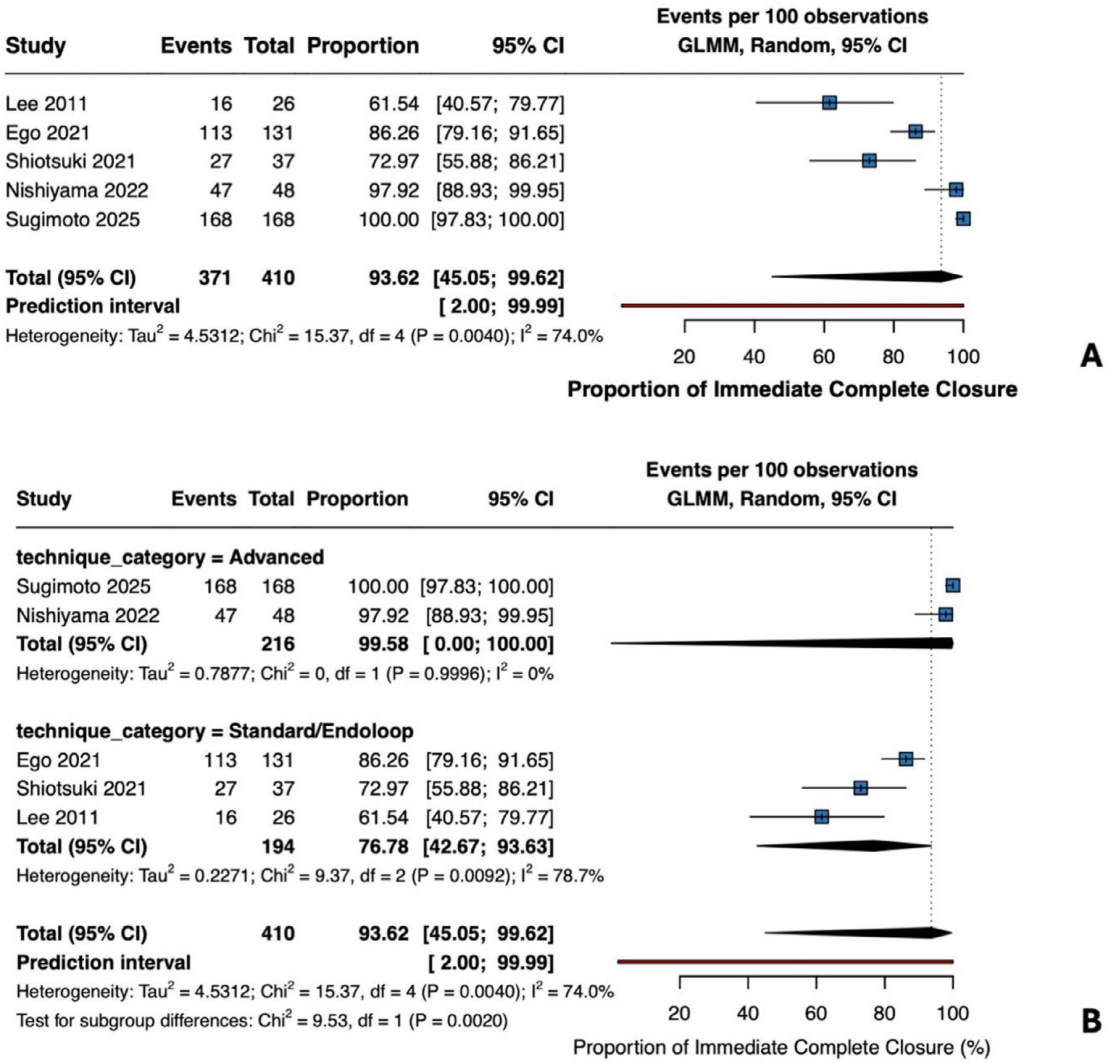
Immediate complete closure rate (comparative studies) (A) Single‐arm meta‐analysis of the immediate complete closure rate across five comparative studies reporting this outcome. Pooled proportion 93.6% (95% CI 45.0%–99.6%), *I* ^2^ = 74% (371/410 procedures). (B) By technique: Advanced techniques (ROLM, E‐LOC; 216 procedures) achieved 99.6% (95% CI 97.8%–100%), *I* ^2^ = 0%. Standard approaches (194 procedures) achieved 76.8% (95% CI 42.7%–93.6%), *I* ^2^ = 79%. Test for subgroup differences: χ^2^ = 9.53, df = 1, *p* = 0.002.

Five comparative studies provided data (Figure 6). Pooled complete closure was 93.6% (95% CI 45.0–99.6%; *I* ^2^ = 74%; 371/410 procedures). Advanced techniques achieved 99.6% (95% CI 97.8%–100%; *I* ^2^ = 0%), while standard approaches showed 76.8% (95% CI 42.7–93.6%; *I* ^2^ = 79%). Subgroup difference was significant (*p* = 0.002), confirming the superior feasibility of advanced techniques.

#### 3.9.2 Sustained Closure at Second‐Look Endoscopy

**FIGURE 7:**
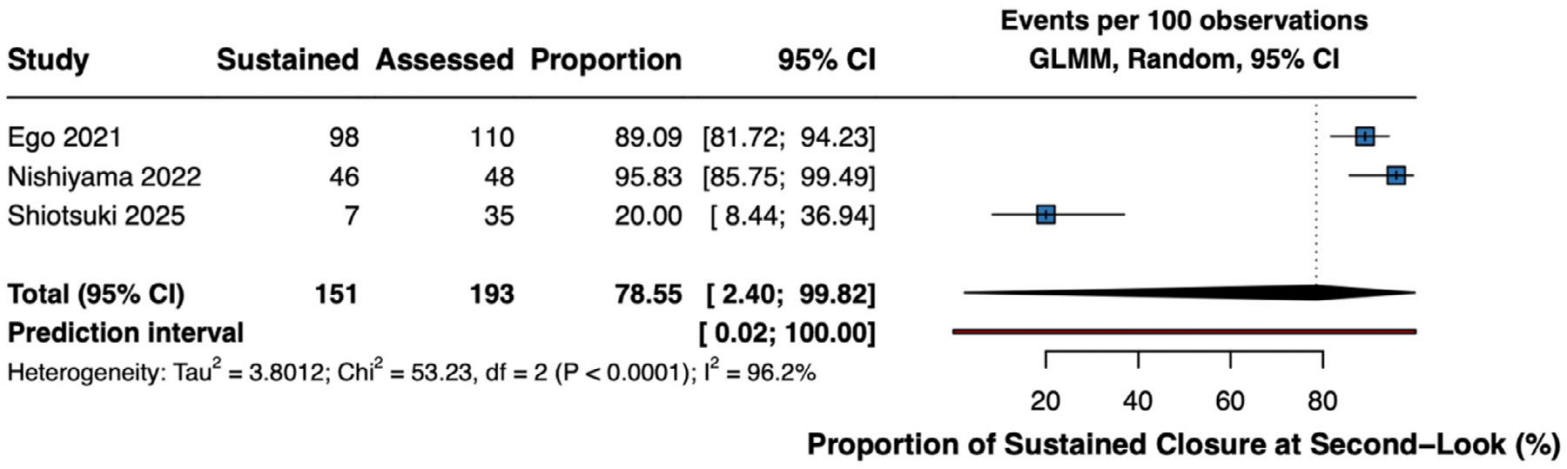
Sustained closure at second‐look endoscopy (comparative studies) Single‐arm meta‐analysis of sustained closure rate at second‐look endoscopy (POD 2–7) across three comparative studies (*n* = 193 procedures). Pooled proportion 78.6% (95% CI 2.45–99.8%), I^2^ = 96%. Extreme heterogeneity precludes reliable interpretation; results should be interpreted cautiously.

Three studies reported sustained closure data (193 procedures) (Figure 7) [16, 36, 39]. Pooled proportion maintaining closure at follow‐up was 78.6% (95% CI 2.4–99.8%; *I* ^2^ = 96%), with high heterogeneity reflecting technique differences. The extreme inconsistency (*I* ^2^ = 96%) precludes reliable interpretation of this outcome; the prediction interval spans from near‐complete failure to universal success, indicating that the true effect in any given population cannot be reliably predicted.

#### 3.9.3 Closure Procedure Time

**FIGURE 8:**
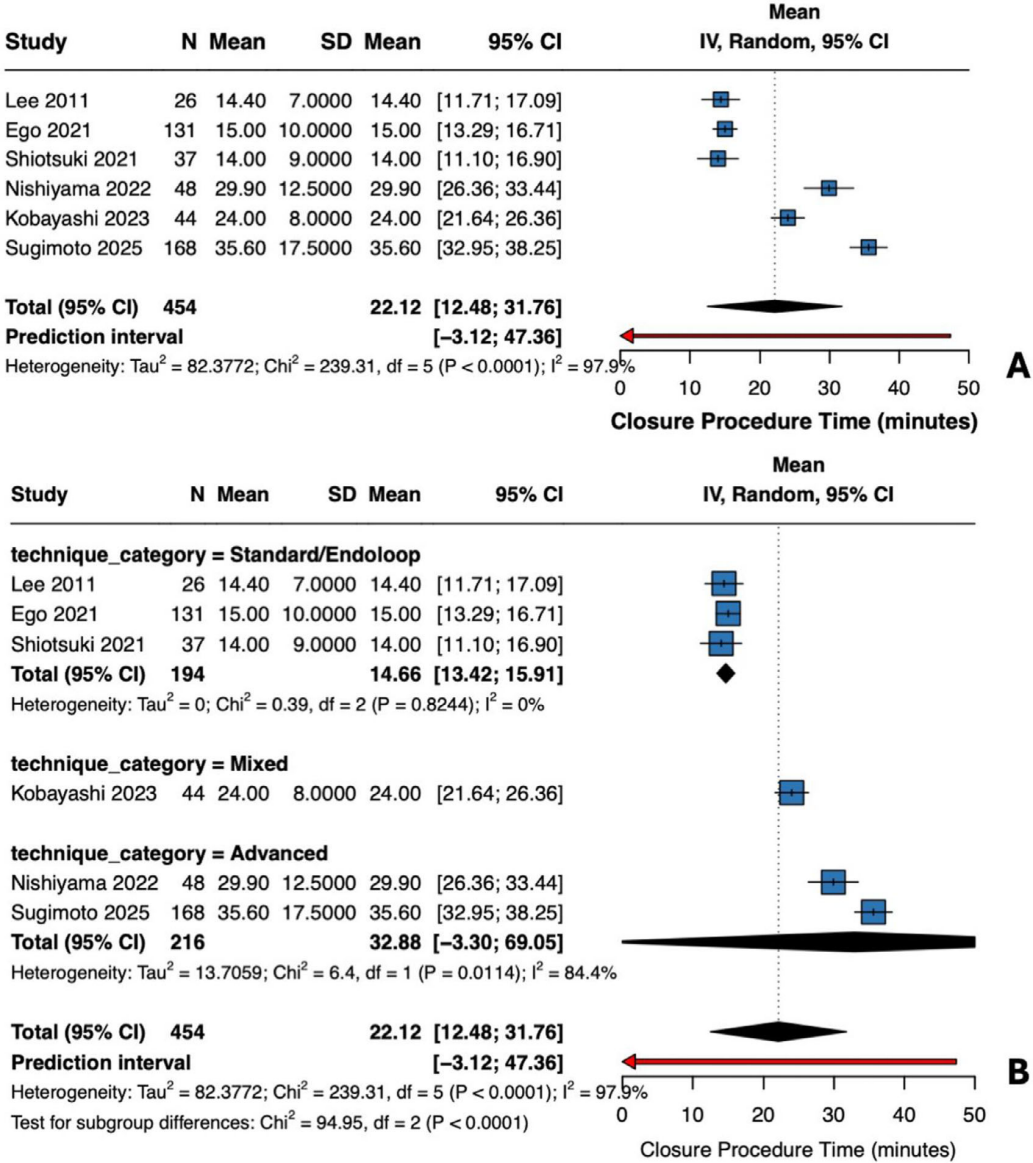
Closure procedure time (comparative studies): (A) Single‐arm meta‐analysis of closure procedure time across six comparative studies (*N* = 454 procedures). Pooled mean 22.1 min (95% CI 12.5–31.8), *I* ^2^ = 97.9%. Individual means: Lee 2011 14.4 min, Ego 2021 15.0 min, Shiotsuki 2021 14.0 min, Nishiyama 2022 29.9 min, Kobayashi 2023 24.0 min, Sugimoto 2025 35.6 min. (B) Subgroup by technique: Standard/Endoloop (three studies, *N* = 194): 14.7 min (95% CI 13.4–15.9), *I* ^2^ = 0%. Advanced techniques (2 studies, *N* = 216): 32.9 min (95% CI −3.3 to 69.1), *I* ^2^ = 84.4%. Test for subgroup differences: χ^2^ = 94.95, df = 2, *p* < 0.0001. Extreme heterogeneity reflects genuine technique differences.

Six studies provided time data (Figure 8) [16, 34, 35, 36, 37, 39]. Pooled mean was 22.1 min (95% CI 12.5–31.8; *I* ^2^ = 97.9%). Standard methods required 14.7 min (95% CI 13.4–15.9; *I* ^2^ = 0%), while advanced techniques required 32.9 min (*I* ^2^ = 84.4%). Subgroup difference was significant (*p* < 0.0001), confirming technique‐dependent procedural duration. The extreme heterogeneity (*I* ^2^ = 98%) reflects genuine differences in technique complexity rather than measurement inconsistency.

#### 3.9.4 Closure‐Related Adverse Events

Four comparative studies explicitly reported closure‐related adverse events in the closure arm [16, 34, 35, 36]. No adverse events attributable to the closure procedure or device were documented. The pooled proportion from generalized linear mixed model analysis was 0.00% (95% CI approximating 0.00%–0.01%), indicating excellent procedural safety across techniques when performed by experienced operators.

### 3.10 Expanded Technical Feasibility Analysis

Incorporating eight additional single‐arm studies [15, 40, 41, 42, 43, 44, 45, 46] (Table S1) enhanced the precision of technical outcome estimates. Across 13 closure cohorts (634 procedures), pooled immediate complete closure was 95.4% (95% CI 83.9%–98.8%; I^2^ = 48.5%), with advanced techniques achieving 98.7% versus 84.3% for standard approaches (*p* = 0.001; Figure S7). Sustained closure at second‐look (seven studies, 280 procedures) was 89.6% (95% CI 44.5–98.9%; *I* ^2^ = 88.6%; Figure S8), though extreme heterogeneity limits interpretation. Pooled procedure time was 24.9 min (95% CI 17.4–32.3; *I* ^2^ = 98.1%), with significant technique‐dependent variation (Figure S9). Closure‐related adverse events were rare (0.31%; 2/521 procedures; Figure S10).

### 3.11 Publication Bias and Certainty of Evidence

Funnel plot for the primary outcome showed some asymmetry with potential small‐study effects (Figure S11), though alternative explanations, including true heterogeneity by technique, remain plausible. With only nine studies, Egger's test lacks adequate power, so the absence of statistical significance (*p* = 0.25) should not be interpreted as the absence of publication bias. GRADE assessment classified evidence certainty as low for clinically significant delayed bleeding (Table 3), reflecting observational study predominance and inconsistency (*I* ^2^ = 65%). Evidence certainty for immediate complete closure was low, primarily due to nonrandomized study designs and substantial between‐study variability; nevertheless, immediate closure rates were consistently high in cohorts using advanced techniques. Evidence certainty was very low for sustained closure due to severe inconsistency (*I* ^2^ = 96%) and imprecision.

**TABLE 3:** GRADE summary of findings.

Outcome	No. of studies	No. of procedures/patients	Effect estimate (95% CI)^a^	Certainty	Reasons for downgrading
Clinically significant delayed bleeding (primary)	9	2646 patients	RR 0.36 (0.16–0.82)	⊕⊕⊖⊖ Low	Risk of bias^b^, Inconsistency^c^, Publication bias^d^
Delayed perforation	3	1123 patients	Not estimable (0 events)	⊕⊖⊖⊖ Very Low	Risk of bias^b^, Inconsistency^e^, Imprecision^f^
Length of hospital stay	2	976 patients	Not pooled	⊕⊖⊖⊖ Very Low	Risk of bias^b^, Inconsistency^g^, Indirectness^h^, Imprecision^i^
Immediate complete closure	5	410 procedures	93.6% (45.0%–99.6%)	⊕⊕⊖⊖ Low	Risk of bias^b^, Inconsistency^j^
Sustained closure at SLE	3	193 procedures	78.6% (2.4%–99.8%)	⊕⊖⊖⊖ Very Low	Risk of bias^b^, Severe inconsistency^k^, Imprecision^l^
Closure procedure time	6	454 procedures	22.1 min (12.5–31.8)	⊕⊖⊖⊖ Very Low	Risk of bias^b^, Severe inconsistency^m^
Closure‐related adverse events	4	521 procedures	0.00% (0.00%–0.01%)	⊕⊕⊖⊖ Low	Risk of bias^b^, Imprecision^n^

^a^ CI calculated by the Hartung‐Knapp‐Sidik‐Jonkman method for comparative outcomes; generalized linear mixed model for proportions.

^b^ Serious risk of bias: eight of nine studies were observational; six were judged at serious risk of bias and two at moderate risk, with only one randomized trial at low risk.

^c^ *I* ^2^ = 65%, indicating substantial heterogeneity; prediction interval spans potential harm (0.05–2.65).

^d^ Funnel plot asymmetry noted; Egger's test underpowered with <10 studies.

^e^ Zero events preclude heterogeneity assessment.

^f^ Optimal information size not met; confidence interval not estimable.

^g^ *I* ^2^ = 96%, reflecting different healthcare systems and practice patterns rather than true clinical differences.

^h^ Outcome influenced by institutional policies rather than intervention effects.

^i^ Only two studies; confidence interval spans clinically meaningful effects in both directions.

^j^ *I* ^2^ = 74%; prediction interval 2.0–99.99%.

^k^ *I* ^2^ = 96%; prediction interval spans near‐complete failure to universal success.

^l^ Confidence interval 2.4–99.8% precludes reliable interpretation.

^m^ I^2^ = 98%; heterogeneity reflects genuine technique complexity differences.

^n^ Rare events with a wide confidence interval.

## 4 Discussion

This systematic review and meta‐analysis suggest that prophylactic mechanical closure may reduce delayed bleeding after gastric ESD. Analysis of nine comparative studies enrolling 2,646 patients showed that closure was associated with reduced delayed bleeding risk (RR 0.36, 95% CI 0.16–0.82; *p* = 0.02), with all individual studies demonstrating directional benefit favoring closure. However, these findings must be interpreted with caution, given the predominantly observational nature of the evidence and the low certainty assigned by GRADE assessment.

If confirmed, the observed association (RR 0.36) would represent a clinically relevant effect, particularly for high‐risk patients where baseline bleeding rates approach 15%–45%. If baseline bleeding risk is 10%, this translates to a number needed to treat of approximately 16 patients to prevent one bleeding episode. For patients on dual antiplatelet therapy where baseline rates may exceed 20%, the absolute risk reduction becomes more substantial. Delayed bleeding carries significant consequences, including emergency endoscopy, blood transfusion, and prolonged hospitalization. The directional consistency across all nine studies—with every individual study showing point estimates favoring closure—supports the plausibility of a beneficial association, though not its confirmation.

The Chen 2022 network meta‐analysis [47], encompassing 2742 lesions across various post‐ESD management strategies, demonstrated that closure techniques may reduce delayed bleeding risk. Our quantitative synthesis extends this assessment by providing pooled effect estimates specifically for mechanical closure across the largest comparative patient sample to date. The Gong 2024 comprehensive review [48] systematically characterized five categories of closure methods, highlighting the technical evolution from simple clip closure to advanced suturing techniques.

Studies of ROLM, E‐LOC, and EHS have individually reported excellent closure success rates exceeding 95%. Our pooled analysis provides comparative context: advanced techniques were associated with lower bleeding rates (RR 0.14) compared with standard approaches (RR 0.62), though this observational difference must be interpreted cautiously given confounding by operator expertise.

Current international guidelines remain equivocal. The ESGE 2022 guideline suggests against routine prophylactic closure while acknowledging potential benefit in selected high‐risk patients [17]. The ASGE 2023 guideline does not provide specific closure recommendations [18]. Japanese guidelines suggest individualized decision‐making [20, 21]. The evidence landscape differs across anatomical sites: the EPOC trial [49] demonstrated significant bleeding reduction with prophylactic closure in colorectal ESD (OR 0.28, 95% CI 0.13–0.60), but no randomized trials evaluating modern advanced closure techniques in gastric ESD have been published.

The biological rationale for prophylactic closure centers on physical protection of exposed submucosal vessels during the vulnerable healing period [7]. Post‐ESD ulcers contain arterioles that remain susceptible to acid‐peptic injury and mechanical trauma until re‐epithelialization, typically requiring 2–8 weeks [50]. Mucosal approximation accelerates granulation tissue formation, promotes angiogenesis, and shields vulnerable vessels from gastric acid exposure [47]. Notably, sustained closure may not be a prerequisite for bleeding prevention; even transient approximation during the early high‐risk period (48–72 h) may confer protection by allowing initial clot stabilization—a hypothesis consistent with observed bleeding reduction despite variable sustained closure rates.

The predominantly observational nature of the included studies represents a fundamental limitation. Eight of nine comparative studies were non‐randomized, introducing substantial risk of confounding by indication. Risk of bias assessment revealed that six of nine studies (67%) had a serious overall risk of bias, fundamentally constraining confidence in the pooled effect estimate. The single randomized trial (Lee 2011) predates modern advanced closure techniques. Additionally, in some observational datasets, the ‘closure’ group was defined by achieving complete closure rather than an intention‐to‐treat attempt, which may introduce selection bias if unsuccessful closure attempts were analyzed with controls.

The sensitivity analysis restricted to high‐quality designs (one RCT plus two propensity‐matched studies, 452 patients) yielded a point estimate (RR 0.30) directionally consistent with the primary analysis but with extremely wide confidence intervals (0.01–10.44) and loss of statistical significance (*p* = 0.28). This demonstrates that when analysis is restricted to designs least susceptible to confounding, the evidence does not reach conventional significance thresholds. This could be interpreted as showing no clear evidence of efficacy; alternatively, inadequate statistical power with only three studies provides an explanation for the wide confidence interval.

Pre‐specified subgroup analyses by antithrombotic therapy status and defect size should be interpreted as exploratory rather than confirmatory. The marked difference in efficacy between advanced and standard techniques (interaction p = 0.005) cannot establish a causal advantage of specific devices, as advanced techniques are concentrated in specialized high‐volume centers with experienced operators. Studies demonstrating the efficacy of advanced techniques were conducted predominantly at specialized Japanese academic centers, with operators required to have completed 100–500 gastric ESDs depending on the technique [15, 34, 41]. Real‐world effectiveness in non‐expert settings may differ meaningfully.

The substantial heterogeneity (*I* ^2^ = 65%) warrants careful interpretation. The prediction interval (RR 0.05–2.65) spans both substantial benefit and potential harm. Additional limitations include geographic concentration in Asian centers (8/9 studies), limiting generalizability to non‐Asian populations where ESD outcomes may differ [51], and borderline funnel plot asymmetry.

GRADE assessment rated evidence certainty as low for the primary outcome, reflecting observational study predominance and inconsistency. Evidence certainty was rated very low for sustained closure outcomes due to severe inconsistency (*I* ^2^ = 96%). These ratings indicate that the true effect may be substantially different from observed estimates.

Given the low certainty of evidence and absence of randomized comparisons in high‐risk populations, the present findings do not support immediate guideline revision or strong practice recommendations. These results should inform the prioritization of adequately powered randomized trials. Given the current evidence limitations, clinical decision‐making should weigh suggestive but low‐certainty evidence against procedural considerations, including time, cost, and operator expertise. These findings do not support changes to current guideline recommendations, which appropriately defer to individual clinical judgment.

Future research should prioritize randomized controlled trials comparing advanced closure techniques to no closure in high‐risk populations. Head‐to‐head comparisons of different techniques would identify optimal approaches. Implementation studies characterizing learning curves and technique generalizability beyond expert centers would assess real‐world effectiveness. Cost‐effectiveness analyses would inform resource allocation.

## 5 Conclusions

This meta‐analysis suggests that prophylactic mechanical closure may reduce delayed bleeding after gastric ESD. However, evidence certainty is low due to observational study predominance (eight of nine studies non‐randomized) and substantial heterogeneity (*I* ^2^ = 65%). Six of nine studies had a serious risk of bias, and sensitivity analysis restricted to high‐quality designs did not maintain statistical significance.

The association appears directionally consistent across patient subgroups in exploratory analyses, though wide confidence intervals preclude definitive conclusions about effect consistency across antithrombotic status or defect size categories. Exploratory analyses observed differences in effect estimates across closure techniques; however, these findings are confounded by operator expertise and center volume, precluding causal inference regarding specific devices or methods.

Given the low certainty evidence, these findings should be considered hypothesis‐generating pending confirmatory randomized trial data. The present evidence does not support immediate guideline revision but does support prioritization of adequately powered randomized trials in high‐risk populations. Clinical decision‐making should weigh the observed association against procedural considerations, recognizing substantial uncertainty.

## Author Contributions

**Hariruk Yodying**: Conception, design, data collection, analysis, interpretation, manuscript drafting, and critical revision. **Vichit Viriyaroj** and **Thammanij Rookkachart**: Data collection, analysis, and critical revision. **Thana Boonsinsukh** and **Anuwat Chartkitcharoen**: Data collection and quality assessment. **Wannakorn Prapasajchavet**, **Patcharaon Petchkaewkul**, and **Natchanok Mekrugsakit**: Data extraction and statistical analysis. **Ratchanon Laojanun** and **Suun Sathornviriyapong**: Study selection, bias assessment, and critical revision.

## Funding

This systematic review and meta‐analysis received no specific grant from any funding agency in the public, commercial, or not‐for‐profit sectors. All work was conducted using institutional library access and statistical software provided by Srinakharinwirot University.

## Ethics Statement

Per institutional policy (Srinakharinwirot University Announcement 8/2567, August 21, 2024), this systematic review of published literature did not require ethics approval. The principal investigator self‐certified exemption eligibility. The study followed PRISMA 2020 guidelines and the Declaration of Helsinki.

## Conflicts of Interest

The authors declare no conflicts of interest.

## Supporting information




**Table S1**: Characteristics of Eight Additional Single‐Arm Studies: Descriptive table of eight single‐arm feasibility studies included for expanded technical outcome analysis (Maekawa 2015, Goto 2020, Shiotsuki 2025, Yoshida 2021, Kinoshita 2020, Nomura 2023, Akimoto 2022, Goto 2025). Includes study design, sample size, closure technique, antithrombotic status, defect size, second‐look endoscopy timing, closure time, immediate complete closure rate, sustained closure rate, and closure‐related adverse events.
**Table S2**: Risk of Bias Assessment Cochrane Risk of Bias 2.0 assessment for randomized controlled trials and ROBINS‐I assessment for non‐randomized studies. Domains assessed include bias from the randomization process, deviations from intended interventions, missing outcome data, outcome measurement, and selection of reported results. Individual study assessments and summary judgments are provided. Six of nine studies (67%) were judged to have a serious risk of bias, primarily due to confounding by indication.
**Figure S1**: Sensitivity Analysis Restricted to High‐Quality Studies Forest plot comparing prophylactic closure versus no closure for clinically significant delayed bleeding, restricted to one randomized controlled trial (Lee 2011) and two propensity score‐matched cohorts (Sugimoto 2025, Kobayashi 2023), total 452 patients (226 closure, 226 control). Random‐effects pooled risk ratio (RR) with Hartung‐Knapp‐Sidik‐Jonkman adjustment: 0.30 (95% CI 0.01–10.44), T = 1.45, df = 2, *p* = 0.28, I^2^ = 30%. Heterogeneity: τ^2^ = 0.62 (95% CI 0.00→100), χ^2^ = 2.86, df = 2, *p* = 0.24. The effect estimate maintained directional benefit, but the wide CI reflects limited statistical power with only three high‐quality studies. A loss of statistical significance reflects limited power rather than the absence of an effect, though this finding demonstrates that the primary analysis relies heavily on observational studies with a serious risk of bias.
**Figure S2**: Sensitivity Analysis Excluding Historical Control Study Forest plot of eight studies (*n* = 2548 patients: 1080 closure, 1,468 control) after excluding Nishiyama 2022 historical control study with serious risk of bias from temporal confounding. Random‐effects pooled RR with HKSJ adjustment: 0.40 (95% CI 0.18–0.91), T = 2.65, df = 7, *p* = 0.03, I^2^ = 65%. Heterogeneity: τ^2^ = 0.61 (95% CI 0.04–4.39), χ^2^ = 20.12, df = 7, *p* = 0.005. Events: 34 in closure versus 146 in control. The effect remained statistically significant despite removing one study, suggesting the historical control design did not drive primary findings.
**Figure S3**: Sensitivity Analysis Using Strict Bleeding Definition Forest plot of eight studies (*n* = 2187 patients: 966 closure, 1,221 control) applying a strict outcome definition requiring documented therapeutic intervention (endoscopic hemostasis, angiographic embolization, surgery, or transfusion). Random‐effects pooled RR with HKSJ adjustment: 0.35 (95% CI 0.13–0.95), T = 2.49, df = 7, *p* = 0.04, I^2^ = 70%. Heterogeneity: τ^2^ = 0.93 (95% CI 0.08–6.54), χ^2^ = 23.14, df = 7, *p* = 0.002. Excluded Wang 2023 for unclear intervention requirement; 29 bleeding events in closure versus 132 in control. Statistical significance maintained with effect estimate consistent with primary analysis.
**Figure S4**: Sensitivity Analysis Excluding Non‐Protocolized Techniques Forest plot of eight studies (*n* = 2511 patients: 1083 closure, 1428 control) after excluding Ramai 2025 using non‐standardized operator‐dependent closure approaches. Random‐effects pooled RR with HKSJ adjustment: 0.31 (95% CI 0.13–0.76), T = 3.09, df = 7, *p* = 0.02, I^2^ = 68%. Heterogeneity: τ^2^ = 0.75 (95% CI 0.05–5.61), χ^2^ = 21.64, df = 7, *p* = 0.003. Events: 31 in closure versus 150 in control. Effect strengthened compared with primary analysis, demonstrating benefit persists across protocolized standardized closure implementations.
**Figure S5**: Sensitivity Analysis Using Alternative Antithrombotic Therapy Categorization Panel A: Exclusive ATA cohorts (Ego 2021, Shiotsuki 2021, Kobayashi 2023, Nishiyama 2022; *n* = 740: 248 closure, 492 control): RR 0.34 (95% CI 0.05–2.48), T = 1.73, df = 3, *p* = 0.18, I^2^ = 67%. Heterogeneity: τ^2^ = 0.94 (95% CI 0.00–23.87), χ^2^ = 8.96, df = 3, *p* = 0.03. Events: 18 in closure versus 79 in control. Panel B: Mixed/Non‐ATA cohorts (Chen 2025, Sugimoto 2025, Wang 2023, Lee 2011, Ramai 2025; *n* = 1906: 880 closure, 1026 control): RR 0.35 (95% CI 0.09–1.31), T = 2.21, df = 4, *p* = 0.09, I^2^ = 58%. Heterogeneity: τ^2^ = 0.56 (95% CI 0.00–12.39), χ^2^ = 9.46, df = 4, *p* = 0.05. Events: 16 in closure versus 77 in control.
**Figure S6**: Length of Hospital Stay—Descriptive Visualization Forest plot displaying length of hospital stay data from two reporting studies. Chen 2025 (*n* = 924: 479 closure, 445 control): mean 8.33 ± 2.22 days versus 8.67 ± 2.96 days, MD −0.34 (95% CI −0.68 to −0.00). Lee 2011 (*n* = 52: 26 closure, 26 control): mean 3.00 ± 0.50 days versus 6.00 ± 2.50 days, MD −3.00 (95% CI −3.98 to −2.02). Extreme heterogeneity (I^2^ = 96%) precludes pooled analysis; marked inconsistency reflects different healthcare systems and temporal practice patterns. Visualization provided for transparency; statistical pooling not performed.
**Figure S7**: Immediate Complete Closure Rate—Expanded Technical Feasibility Panel A: Forest plot of single‐arm proportion meta‐analysis for immediate complete closure rates across 13 closure cohorts (*n* = 634 procedures), pooling nine comparative study closure arms with 4 single‐arm feasibility studies. Random‐effects model using generalized linear mixed model with Freeman‐Tukey double arcsine transformation. Pooled proportion 95.4% (95% CI 83.9%–98.8%), I^2^ = 48.5%. Individual study proportions ranged from 61.5% (Lee 2011 detachable snare+clips) to 100.0% (Sugimoto 2025 reopenable clip‐over‐line method, Akimoto 2022 endoscopic hand suturing, Nomura 2023 reopenable clip‐over‐line method, Goto 2025 endoscopic hand suturing). Panel B: Subgroup forest plot stratifying 13 closure cohorts by technique sophistication. Advanced techniques (reopenable clip‐over‐line method, endoscopic ligation with O‐ring closure, endoscopic hand suturing): 98.7% (95% CI 96.8%–99.5%), I^2^ = 0%, *n* = 281 procedures achieving near‐universal immediate success. Standard techniques (through‐the‐scope clips, endoloop‐based methods): 84.3% (95% CI 68.55%–92.9%), I^2^ = 79%, *n* = 353 procedures with greater variability. Test for subgroup difference: χ^2^ = 20.00, *p* = 0.0012, confirming statistically significant technical superiority of advanced closure systems.
**Figure S8**: Sustained Closure at Second‐Look Endoscopy—Expanded Analysis Panel A: Forest plot of single‐arm proportion meta‐analysis for sustained closure rates at second‐look endoscopy from seven reporting cohorts (*n* = 280 procedures assessed). Random‐effects model using a generalized linear mixed model with logit link. Pooled proportion 89.6% (95% CI 44.5%–98.9%), I^2^ = 88.6%, prediction interval 1.9%–99.97%. Individual proportions ranged from 20.0% (Shiotsuki 2025 flexible endoloop assessed postoperative day 5–7) to 100.0% (Shiotsuki 2021 endoloop, Akimoto 2022 endoscopic hand suturing). Very wide confidence and prediction intervals reflect substantial heterogeneity in second‐look timing (postoperative day 2–7) and closure techniques. Panel B: Subgroup forest plot stratifying 7 cohorts by technique family. Endoloop‐based (Ego 2021, Shiotsuki 2021, Shiotsuki 2025): 78.6% (95% CI 0.07%–99.99%), I^2^ = 72%, *n* = 140, marked heterogeneity driven by assessment timing variation. Endoscopic ligation with O‐ring closure (Nishiyama 2022 only): 95.8% (95% CI 85.8%–99.5%), *n* = 48, the highest sustained rate with early assessment (postoperative day 2–3). Endoscopic hand suturing (Goto 2020, Akimoto 2022, Goto 2025): 89.3% (95% CI 55.97%–98.19%), I^2^ = 0%, *n* = 92, homogeneous across three independent cohorts. Test for subgroup difference: χ^2^ = 1.75, *p* = 0.42, not statistically significant.
**Figure S9**: Closure Procedure Time—Expanded Analysis Panel A: Forest plot displaying weighted mean closure procedure times from 12 studies with extractable time data (*n* = 612 procedures). Random‐effects model for continuous outcomes using inverse‐variance weighting with Hartung‐Knapp adjustment. Pooled mean 24.9 min (95% CI 17.4–32.3), I^2^ = 98.1%. Five studies reported mean ± SD directly; seven provided median with range/IQR, requiring the Wan/Luo method transformation. Individual mean times ranged from 11.0 min (Shiotsuki 2025 FLEXLOOP) to 49.5 min (Goto 2020 endoscopic hand suturing). Goto 2025 provided a mean of 48 min without SD, excluded from pooling. Panel B: Subgroup forest plot stratifying 12 studies by technique family (eight categories). Endoloop‐based (Lee 2011, Ego 2021, Shiotsuki 2021): mean 14.7 min (95% CI 13.4–15.9), I^2^ = 0%. Mixed Clips+E‐LOC (Kobayashi 2023): 24.0 min. E‐LOC (Nishiyama 2022): 29.9 min. ROLM (Sugimoto 2025, Nomura 2023): 32.7 min (95% CI ‐2.8 to 68.3), I^2^ = 91.1%. OTSC‐hybrid (Maekawa 2015): 15.2 min. EHS (Goto 2020, Akimoto 2022): 42.6 min (95% CI ‐43.2 to 128.3), I^2^ = 92.7%. LOCCM (Yoshida 2021): 24.9 min. FLEXLOOP (Shiotsuki 2025): 11.0 min. Test for subgroup differences: χ^2^ = 202.83, df = 7, *p* < 0.0001.
**Figure S10**: Closure‐Related Adverse Events—Expanded Analysis Forest plot of single‐arm proportion meta‐analysis for closure‐related adverse events from 8 reporting closure cohorts (*n* = 521 procedures: four comparative study closure arms, four single‐arm studies). Random‐effects model using generalized linear mixed model with logit link for rare event meta‐analysis. Pooled proportion 0.31% (95% CI 0.02%–4.13%), I^2^ = 0%, τ^2^ = 0.78, prediction interval 0.01%–8.24%. Only two events total: Goto 2020 (1/30, needle puncture bleeding during endoscopic hand suturing) and Goto 2025 (1/43, minor mucosal erosion). Nine additional studies were excluded as “not reported” because they did not explicitly assess closure‐specific adverse events separately from general post‐endoscopic submucosal dissection complications.
**Figure S11**: Funnel Plot for Publication Bias Assessment Funnel plot of nine comparative studies plotting log risk ratio for clinically significant delayed bleeding (x‐axis) against standard error (y‐axis). Vertical line indicates pooled effect under random‐effects model: log RR = ‐1.02 (equivalent to RR 0.36). Egger's regression test for asymmetry: intercept = ‐3.42, z = ‐1.25, *p* = 0.25 (not statistically significant). Visual inspection suggests possible asymmetry with apparent underrepresentation of small studies showing null or harmful effects; however, the statistical test lacks power with only nine studies (a reliable Egger's test typically requires ≥10 studies).

**Appendix S1**: Detailed Search Strategy Comprehensive five‐database systematic search strategy was conducted in October 2025. Total 476 citations identified.

Completed PRISMA 2020 checklist for systematic reviews, indicating the location of each checklist item within the manuscript and supplementary materials. Complete supplementary materials are available online, including PRISMA 2020 checklist, detailed search strategies (Appendix S1), characteristics of additional single‐arm studies (Table S1), risk of bias assessment (Table S2), sensitivity analyses (Figures S1–S5), length of hospital stay (Figure S6), expanded technical feasibility outcomes (Figures S7–S10), and publication bias assessment (Figure S11).

## Data Availability

The systematic review protocol was prospectively registered with PROSPERO (CRD420251172925) and is available at https://www.crd.york.ac.uk/prospero/. Extracted datasets are available from the corresponding author upon reasonable request.
