# Supplementary material for: Prophylactic Mechanical Closure for Preventing Delayed Bleeding after Gastric Endoscopic Submucosal Dissection: A Systematic Review and Meta‐Analysis
Source: DEN Open. 2026 Feb 14;6(1):e70299. doi: 10.1002/deo2.70299 (PMC12906296; doi:10.1002/deo2.70299)
Supplement: Supplementary file 1 — Table S1: Characteristics of Eight Additional Single‐Arm Studies: Descriptive table of eight single‐arm feasibility studies included for expanded technical outcome analysis (Maekawa 2015, Goto 2020, Shiotsuki 2025, Yoshida 2021, Kinoshita 2020, Nomura 2023, Akimoto 2022, Goto 2025). Includes study design, sample size, closure technique, antithrombotic status, defect size, second‐look endoscopy timing, closure time, immediate complete closure rate, sustained closure rate, and closure‐related adverse events. Table S2: Risk of Bias Assessment Cochrane Risk of Bias 2.0 assessment for randomized controlled trials and ROBINS‐I assessment for non‐randomized studies. Domains assessed include bias from the randomization process, deviations from intended interventions, missing outcome data, outcome measurement, and selection of reported results. Individual study assessments and summary judgments are provided. Six of nine studies (67%) were judged to have a serious risk of bias, primarily due to confounding by indication. Figure S1: Sensitivity Analysis Restricted to High‐Quality Studies Forest plot comparing prophylactic closure versus no closure for clinically significant delayed bleeding, restricted to one randomized controlled trial (Lee 2011) and two propensity score‐matched cohorts (Sugimoto 2025, Kobayashi 2023), total 452 patients (226 closure, 226 control). Random‐effects pooled risk ratio (RR) with Hartung‐Knapp‐Sidik‐Jonkman adjustment: 0.30 (95% CI 0.01–10.44), T = 1.45, df = 2, p = 0.28, I2 = 30%. Heterogeneity: τ2 = 0.62 (95% CI 0.00→100), χ2 = 2.86, df = 2, p = 0.24. The effect estimate maintained directional benefit, but the wide CI reflects limited statistical power with only three high‐quality studies. A loss of statistical significance reflects limited power rather than the absence of an effect, though this finding demonstrates that the primary analysis relies heavily on observational studies with a serious risk of bias. Figure S2: Sensitivity Analysis Exclu [file DEO2-6-e70299-s002.docx]

**SUPPLEMENTARY MATERIALS**

**Prophylactic Mechanical Closure to Prevent Delayed Bleeding After Gastric Endoscopic Submucosal Dissection: A Systematic Review and Meta-analysis**

**Supplementary Tables**

**Supplementary Table S1: Characteristics of Eight Additional Single-Arm Studies**

| **Study (Year)** | **Country** | **Design** | **N** | **Technique Family** | **AT Status** | **Defect Size** | **SLE Timing** | **Closure Time (min)** | **Immediate Complete Closure** | **Sustained Closure at SLE** | **CRAEs** |
| --- | --- | --- | --- | --- | --- | --- | --- | --- | --- | --- | --- |
| Maekawa 2015 | Japan | Retrospective | 12 | OTSC-hybrid | NR | NR | NR | 15.18±8.01ᵃ | 11/12 (91.7%) | NR | NR |
| Goto 2020ᵉ | Japan | Prospective | 30 | EHS | NR | NR | POD 3 | 49.5±16.2 | 29/30 (96.7%) | 25/30 (83.3%) | 1/30 |
| Shiotsuki 2025 | Japan | Prospective | 35 | FLEXLOOP+clips | NR | NR | POD 5-7 | 11 (8-30)ᵇ | 31/35 (88.6%) | 7/35 (20.0%) | 0/35 |
| Yoshida 2021 | Japan | Prospective | 19ᶜ | LOCCM | NR | NR | NR | 24.9±10.9 | 8/10 (80.0%)ᶜ | NR | NR |
| Kinoshita 2020 | Japan | Retrospective | 22 | Advanced clips | NR | NR | NR | NR | 15/22 (68.2%) | NR | NR |
| Nomura 2023 | Japan | Prospective | 50 | ROLM | NR | NR | NR | 30 (14-35)ᵇ | 50/50 (100%) | NR | NR |
| Akimoto 2022 | Japan | Prospective | 22 | EHS | AT-only | NR | Follow-up | 36 (24-60)ᵇ | 22/22 (100%) | 22/22 (100%) | 0/22 |
| Goto 2025ᵉ | Japan | Phase II | 43 | EHS | NR | NR | POD 3 | 48±19 | 43/43 (100%) | 35/40 (87.5%)ᵈ | 1/43 |

**Footnotes:** ᵃ Calculated from individual case data (n=11 with complete time data), ᵇ Median (range), ᶜ Stomach subset only (10/19 total), ᵈ Excludes 2 patients with bleeding before SLE and 1 cancelled case, ᵉ Goto 2020 (Phase I pilot) and Goto 2025 (Phase II expanded) are sequential studies from the same group. Phase I established feasibility and safety in general ESD patients. Phase II evaluated efficacy specifically in high-risk patients on continued antithrombotic therapy.

**Abbreviations:** AT, antithrombotic therapy; CRAEs, closure-related adverse events; EHS, endoscopic hand suturing; FLEXLOOP, flexible endoloop; LOCCM, line-assisted complete closure method; NR, not reported; OTSC, over-the-scope clip; POD, postoperative day; ROLM, reopenable clip-over-the-line method; SLE, second-look endoscopy

**Supplementary Table S2: Risk of Bias Assessment Summary**

| **Study** | **Overall Risk of Bias** | **Key Domains of Concern** |
| --- | --- | --- |
| **Randomized Trial (RoB 2)** |  |  |
| Lee 2011 | Low | Blinding not possible; otherwise low risk across domains |
| **Observational Studies (ROBINS-I)** |  |  |
| Sugimoto 2025 | Moderate | Unmeasured confounding despite PSM; selection of participants |
| Kobayashi 2023 | Moderate | Unmeasured confounding despite PSM; intervention classification |
| Chen 2025 | Serious | Confounding by indication; selection bias in multicenter design |
| Ego 2021 | Serious | Confounding; selection of participants; missing outcome data |
| Shiotsuki 2021 | Serious | Confounding; retrospective design; potential selection bias |
| Wang 2023 | Serious | Confounding; outcome measurement; selective reporting |
| Ramai 2025 | Serious | Confounding; non-standardized intervention; missing data |
| Nishiyama 2022 | Serious | Temporal confounding (historical control); no adjustment |

**Abbreviations:** PSM, propensity score matching; RoB 2, Revised Cochrane Risk of Bias tool; ROBINS-I, Risk of Bias in Non-randomised Studies of Interventions

Cochrane Risk of Bias 2.0 assessment for randomized controlled trials and ROBINS-I assessment for non-randomized studies. Domains assessed include bias from randomization process, deviations from intended interventions, missing outcome data, outcome measurement, and selection of reported results. Individual study assessments and summary judgments provided. Six of nine studies (67%) were judged to have serious risk of bias, primarily due to confounding by indication.

**Supplementary Figures**

**Supplementary Figure S1. Sensitivity Analysis Restricted to High-Quality Studies**


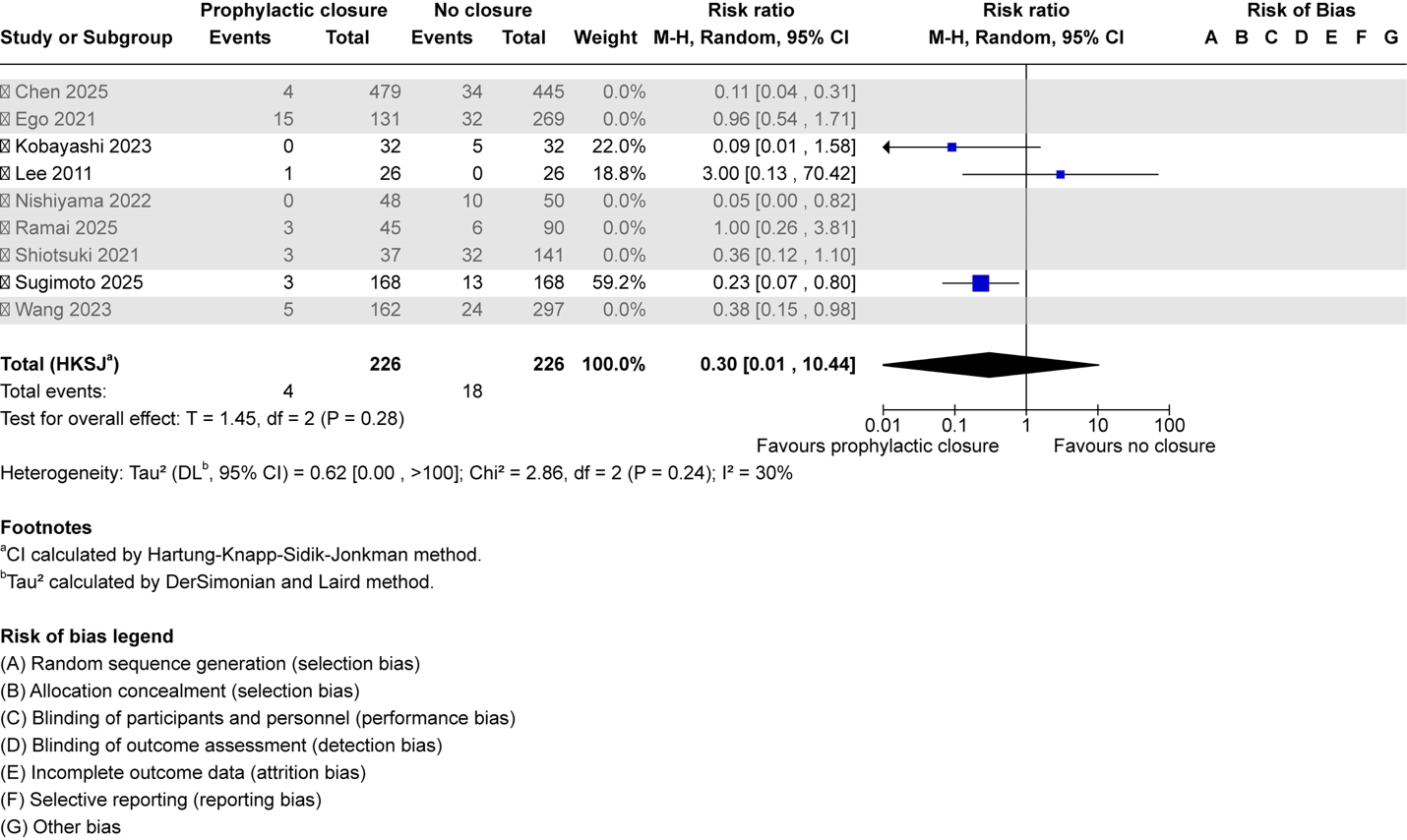


Forest plot comparing prophylactic closure versus no closure for clinically significant delayed bleeding, restricted to one randomized controlled trial (Lee 2011) and two propensity score-matched cohorts (Sugimoto 2025, Kobayashi 2023), total 452 patients (226 closure, 226 control). Random-effects pooled risk ratio (RR) with Hartung-Knapp-Sidik-Jonkman adjustment: 0.30 (95% CI 0.01-10.44), T=1.45, df=2, p=0.28, I²=30%. Heterogeneity: τ²=0.62 (95% CI 0.00->100), Chi²=2.86, df=2, p=0.24. Effect estimate maintained directional benefit but wide CI reflects limited statistical power with only 3 high-quality studies. A loss of statistical significance reflects limited power rather than the absence of an effect, though this finding demonstrates that the primary analysis relies heavily on observational studies with serious risk of bias.

**Supplementary Figure S2. Sensitivity Analysis Excluding Historical Control Study**


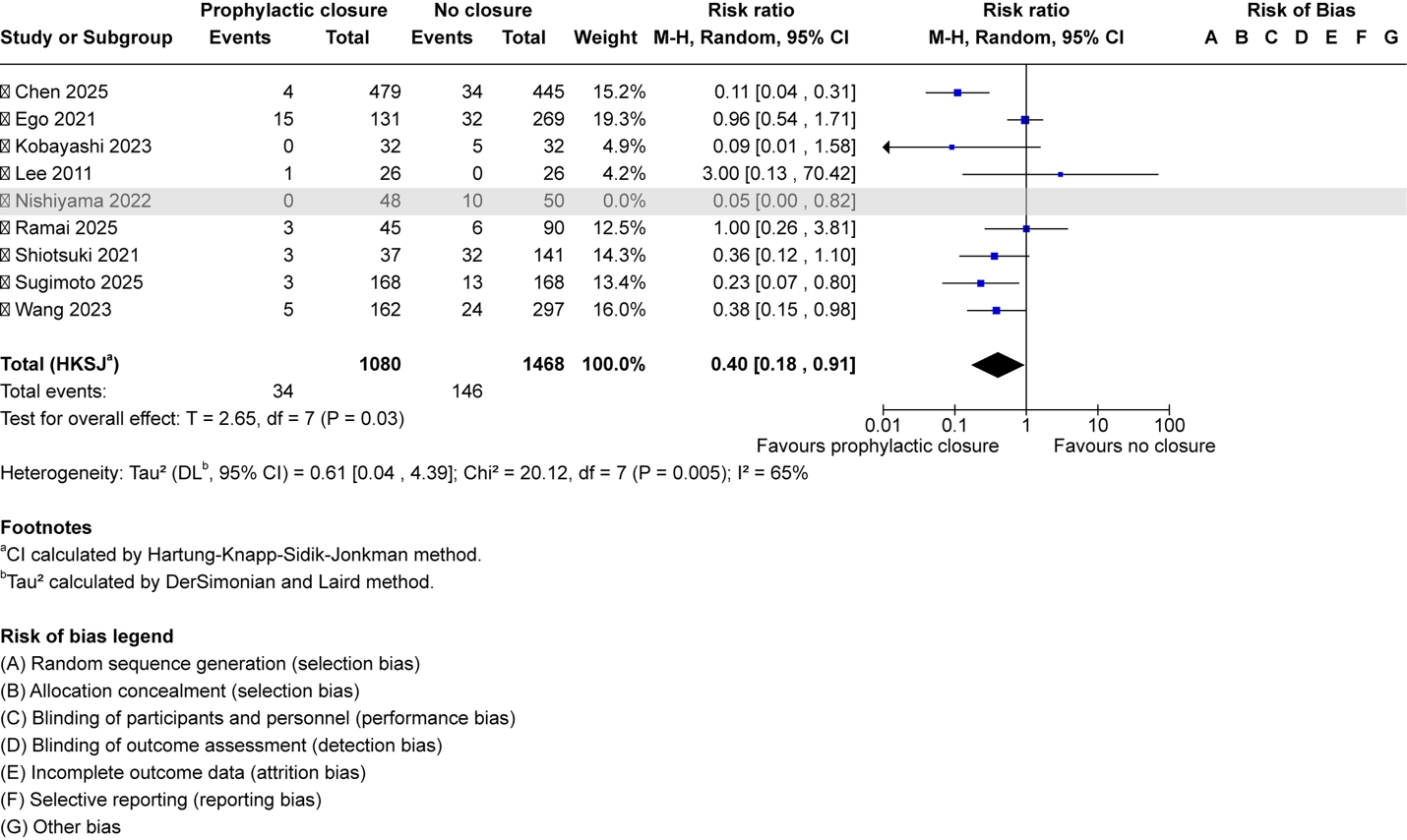


Forest plot of eight studies (n=2,548 patients: 1,080 closure, 1,468 control) after excluding Nishiyama 2022 historical control study with serious risk of bias from temporal confounding. Random-effects pooled RR with HKSJ adjustment: 0.40 (95% CI 0.18-0.91), T=2.65, df=7, p=0.03, I²=65%. Heterogeneity: τ²=0.61 (95% CI 0.04-4.39), Chi²=20.12, df=7, p=0.005. Events: 34 in closure versus 146 in control. Effect remained statistically significant despite removing one study, suggesting historical control design did not drive primary findings.

**Supplementary Figure S3. Sensitivity Analysis Using Strict Bleeding Definition**


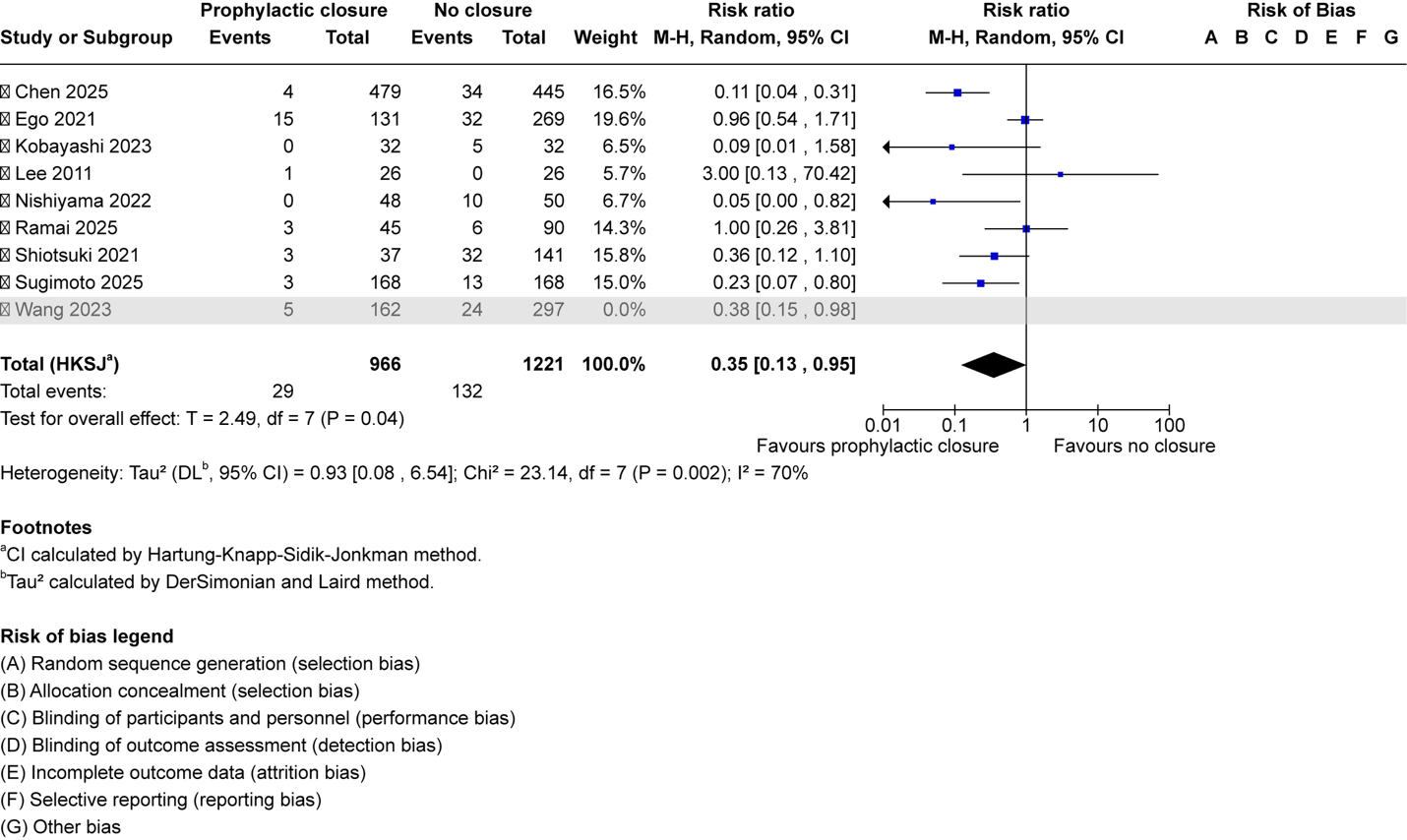


Forest plot of eight studies (n=2,187 patients: 966 closure, 1,221 control) applying strict outcome definition requiring documented therapeutic intervention (endoscopic hemostasis, angiographic embolization, surgery, or transfusion). Random-effects pooled RR with HKSJ adjustment: 0.35 (95% CI 0.13-0.95), T=2.49, df=7, p=0.04, I²=70%. Heterogeneity: τ²=0.93 (95% CI 0.08-6.54), Chi²=23.14, df=7, p=0.002. Excluded Wang 2023 for unclear intervention requirement; 29 bleeding events in closure versus 132 in control. Statistical significance maintained with effect estimate consistent with primary analysis.

**Supplementary Figure S4. Sensitivity Analysis Excluding Non-Protocolized Techniques**


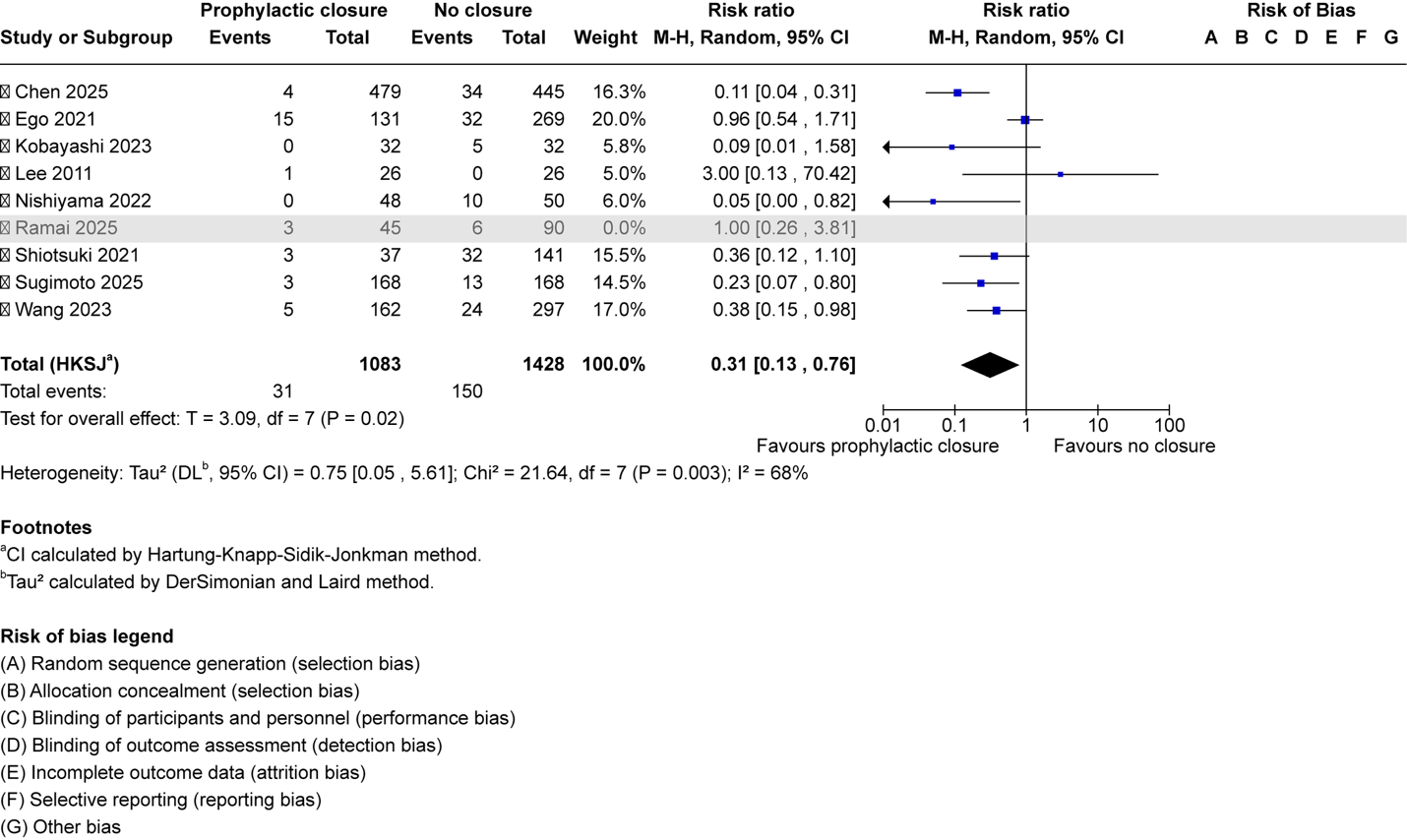


Forest plot of eight studies (n=2,511 patients: 1,083 closure, 1,428 control) after excluding Ramai 2025 using non-standardized operator-dependent closure approaches. Random-effects pooled RR with HKSJ adjustment: 0.31 (95% CI 0.13-0.76), T=3.09, df=7, p=0.02, I²=68%. Heterogeneity: τ²=0.75 (95% CI 0.05-5.61), Chi²=21.64, df=7, p=0.003. Events: 31 in closure versus 150 in control. Effect strengthened compared with primary analysis, demonstrating benefit persists across protocolized standardized closure implementations.

**Supplementary Figure S5. Sensitivity Analysis Using Alternative Antithrombotic Therapy Categorization**

Forest plot re-categorizing nine studies by antithrombotic therapy (ATA) exposure with Hartung-Knapp-Sidik-Jonkman adjustment.


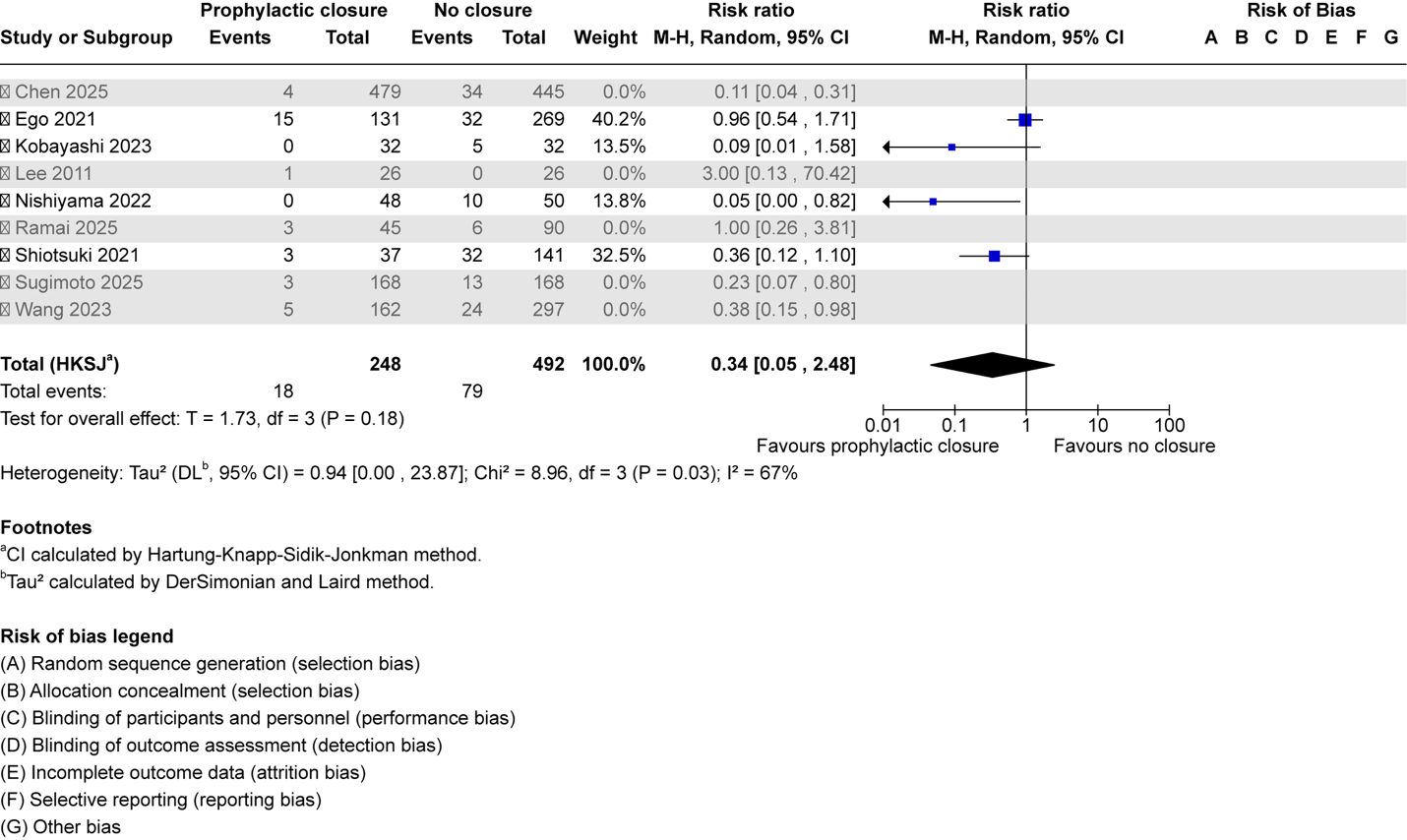


Panel A: Exclusive ATA cohorts (Ego 2021, Shiotsuki 2021, Kobayashi 2023, Nishiyama 2022; n=740: 248 closure, 492 control): RR 0.34 (95% CI 0.05-2.48), T=1.73, df=3, p=0.18, I²=67%. Heterogeneity: τ²=0.94 (95% CI 0.00-23.87), Chi²=8.96, df=3, p=0.03. Events: 18 in closure versus 79 in control.


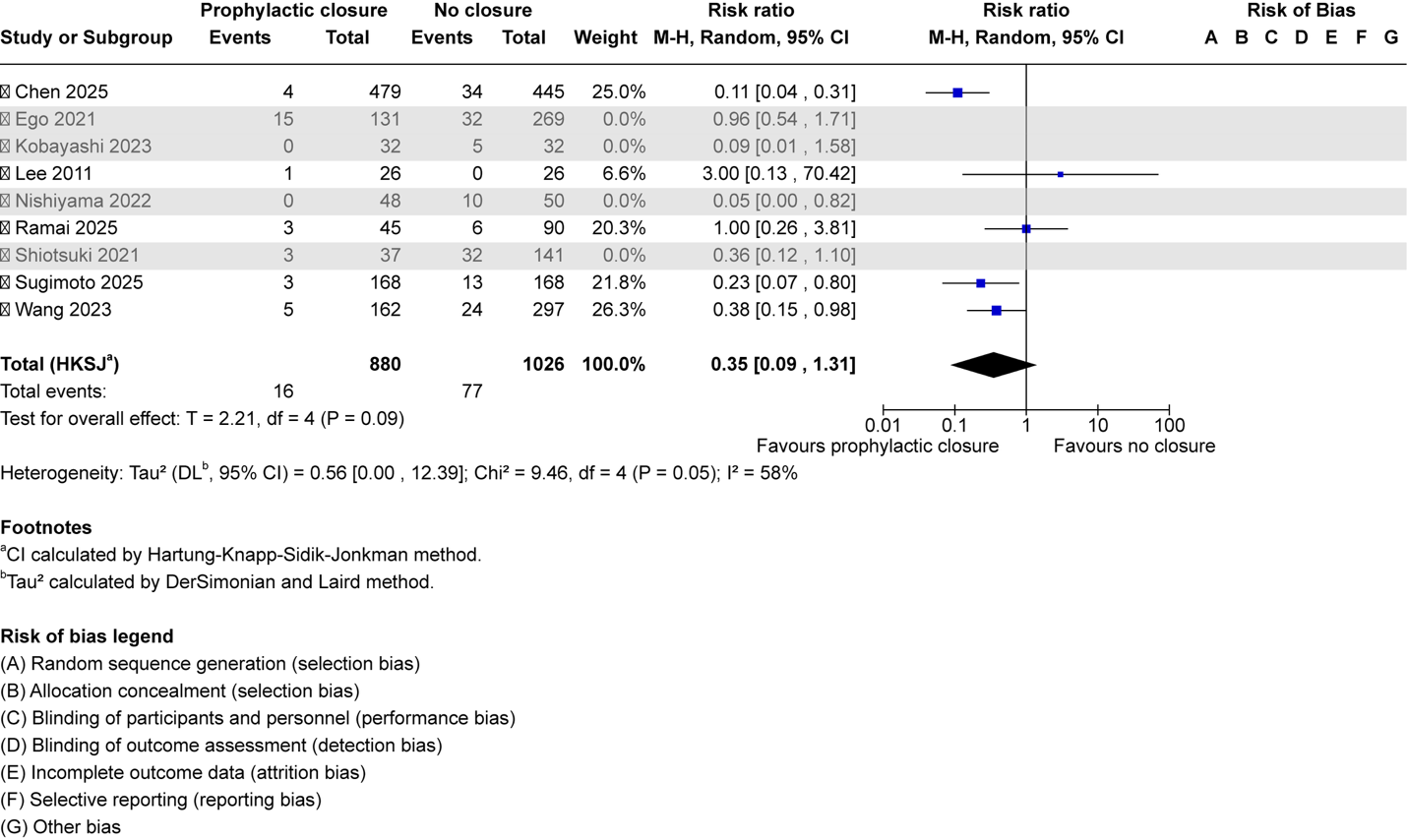


Panel B**:** Mixed/Non-ATA cohorts (Chen 2025, Sugimoto 2025, Wang 2023, Lee 2011, Ramai 2025; n=1,906: 880 closure, 1,026 control): RR 0.35 (95% CI 0.09-1.31), T=2.21, df=4, p=0.09, I²=58%. Heterogeneity: τ²=0.56 (95% CI 0.00-12.39), Chi²=9.46, df=4, p=0.05. Events: 16 in closure versus 77 in control.

**Supplementary Figure S6. Length of Hospital Stay - Descriptive Visualization**

**
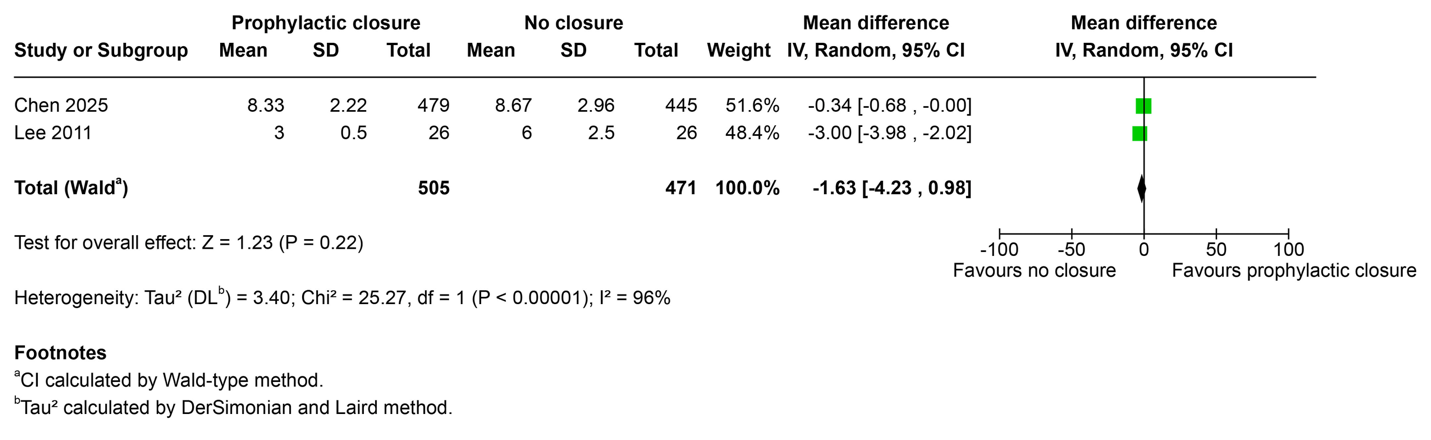
**

Forest plot displaying length of hospital stay data from two reporting studies. Chen 2025 (n=924: 479 closure, 445 control): mean 8.33±2.22 days versus 8.67±2.96 days, MD −0.34 (95% CI −0.68 to −0.00). Lee 2011 (n=52: 26 closure, 26 control): mean 3.00±0.50 days versus 6.00±2.50 days, MD −3.00 (95% CI −3.98 to −2.02). Extreme heterogeneity (I²=96%) precludes pooled analysis; marked inconsistency reflects different healthcare systems and temporal practice patterns. Visualization provided for transparency; statistical pooling not performed.

**Supplementary Figure S7. Immediate Complete Closure Rate - Expanded Technical Feasibility**

**S7_Panel A. Immediate Complete Closure - Overall Analysis**

**
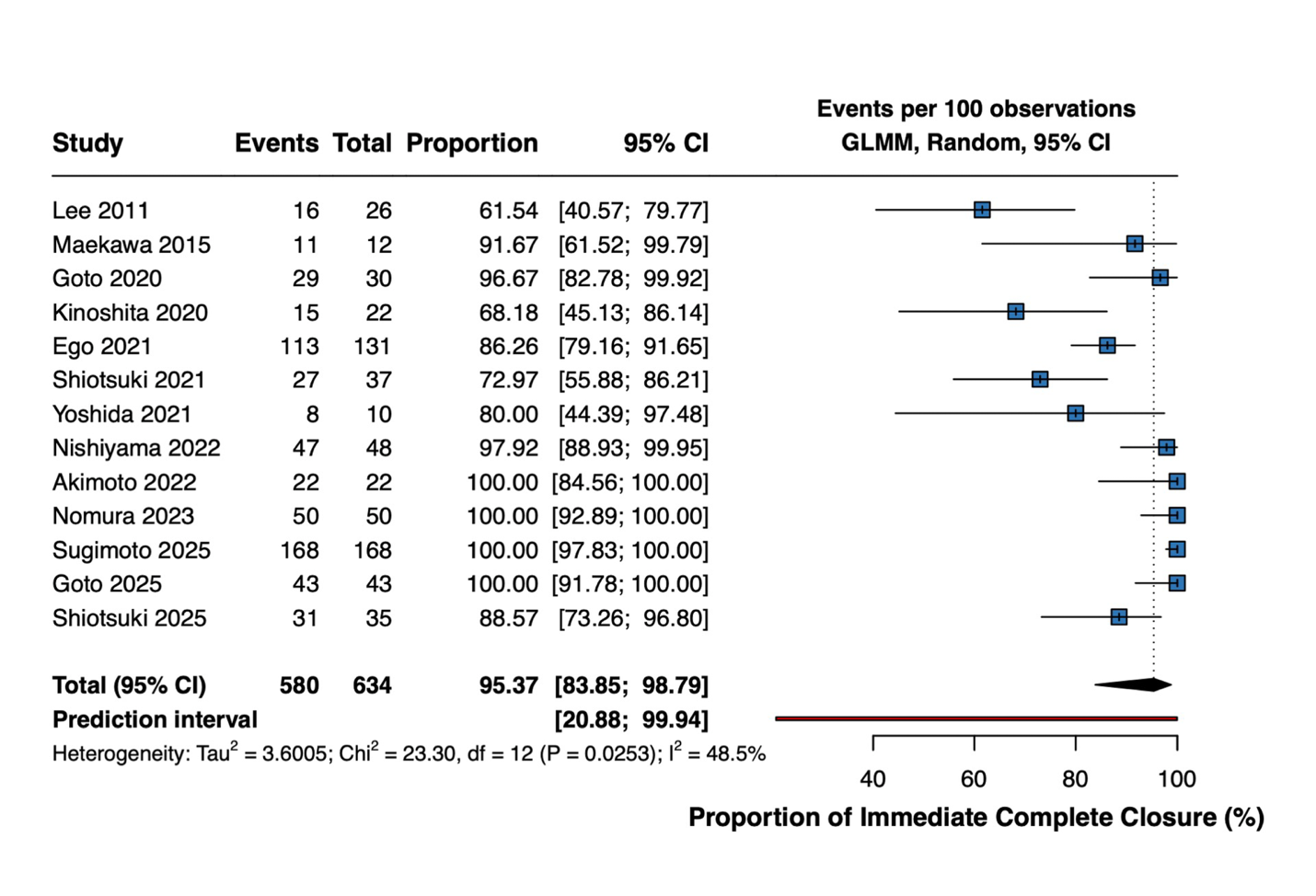
**

Panel A: Forest plot of single-arm proportion meta-analysis for immediate complete closure rates across 13 closure cohorts (n=634 procedures) pooling 9 comparative study closure arms with 4 single-arm feasibility studies. Random-effects model using generalized linear mixed model with Freeman-Tukey double arcsine transformation. Pooled proportion 95.4% (95% CI 83.9-98.8%), I²=48.5%. Individual study proportions ranged from 61.5% (Lee 2011 detachable snare+clips) to 100.0% (Sugimoto 2025 reopenable clip-over-line method, Akimoto 2022 endoscopic hand suturing, Nomura 2023 reopenable clip-over-line method, Goto 2025 endoscopic hand suturing).

**S7_Panel B. Immediate Complete Closure by Technique**

**
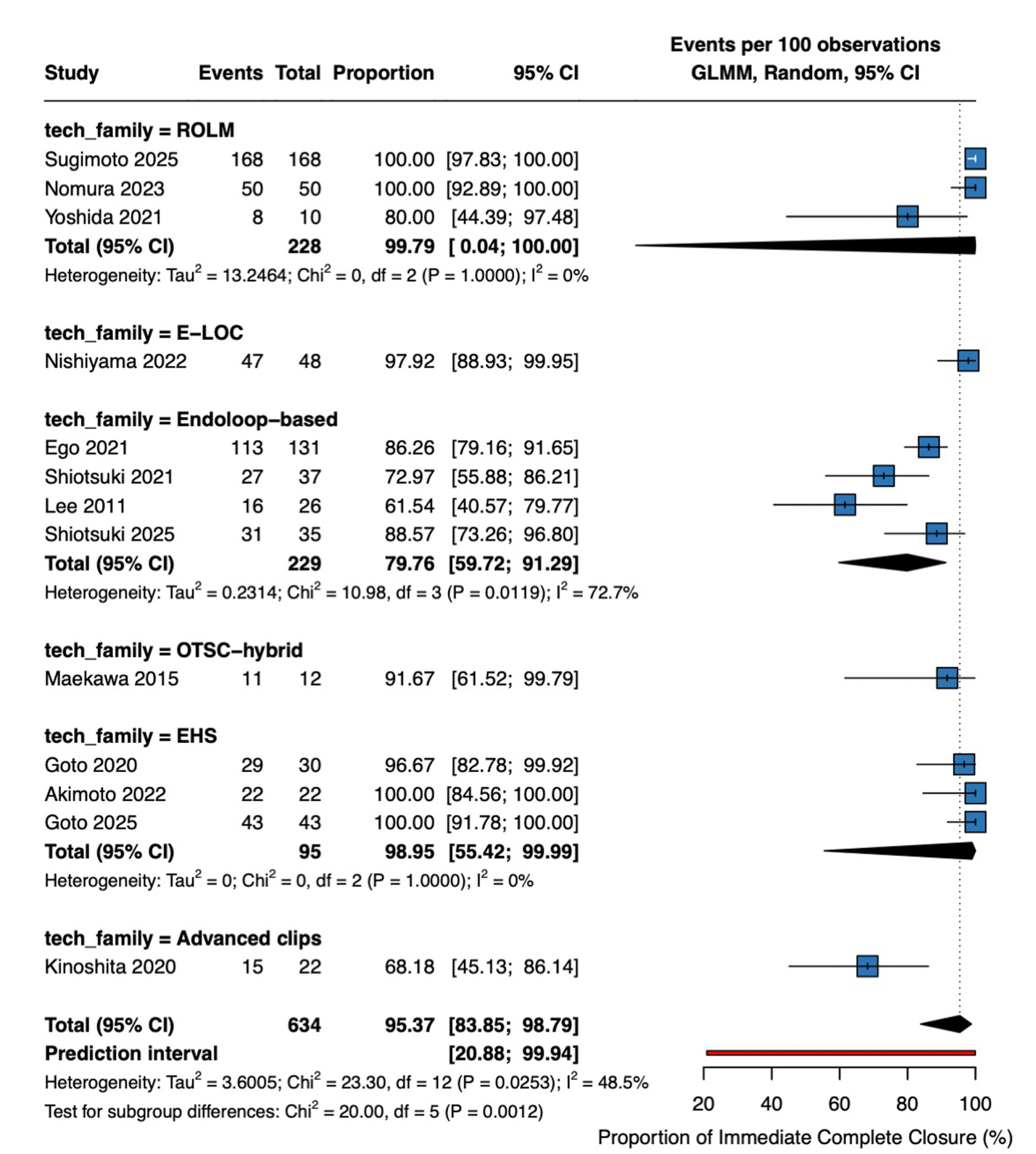
**

Panel B: Subgroup forest plot stratifying 13 closure cohorts by technique sophistication. Advanced techniques (reopenable clip-over-line method, endoscopic ligation with O-ring closure, endoscopic hand suturing): 98.7% (95% CI 96.8-99.5%), I²=0%, n=281 procedures achieving near-universal immediate success. Standard techniques (through-the-scope clips, endoloop-based methods): 84.3% (95% CI 68.5-92.9%), I²=79%, n=353 procedures with greater variability. Test for subgroup difference: χ²=20.00, p=0.0012, confirming statistically significant technical superiority of advanced closure systems.

**Supplementary Figure S8. Sustained Closure at Second-Look Endoscopy - Expanded Analysis**

**
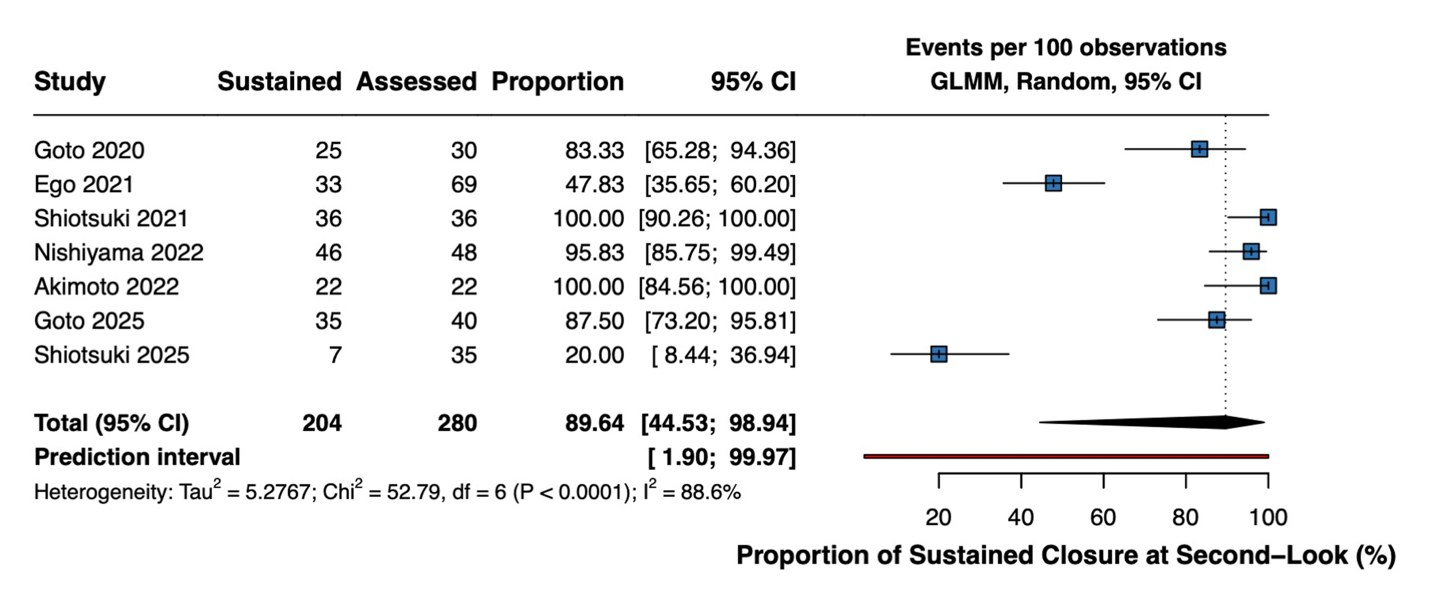
**

Panel A: Forest plot of single-arm proportion meta-analysis for sustained closure rates at second-look endoscopy from 7 reporting cohorts (n=280 procedures assessed). Random-effects model using generalized linear mixed model with logit link. Pooled proportion 89.6% (95% CI 44.5-98.9%), I²=88.6%, prediction interval 1.9-99.97%. Individual proportions ranged from 20.0% (Shiotsuki 2025 flexible endoloop assessed postoperative day 5-7) to 100.0% (Shiotsuki 2021 endoloop, Akimoto 2022 endoscopic hand suturing). Very wide confidence and prediction intervals reflect substantial heterogeneity in second-look timing (postoperative day 2-7) and closure techniques.

**
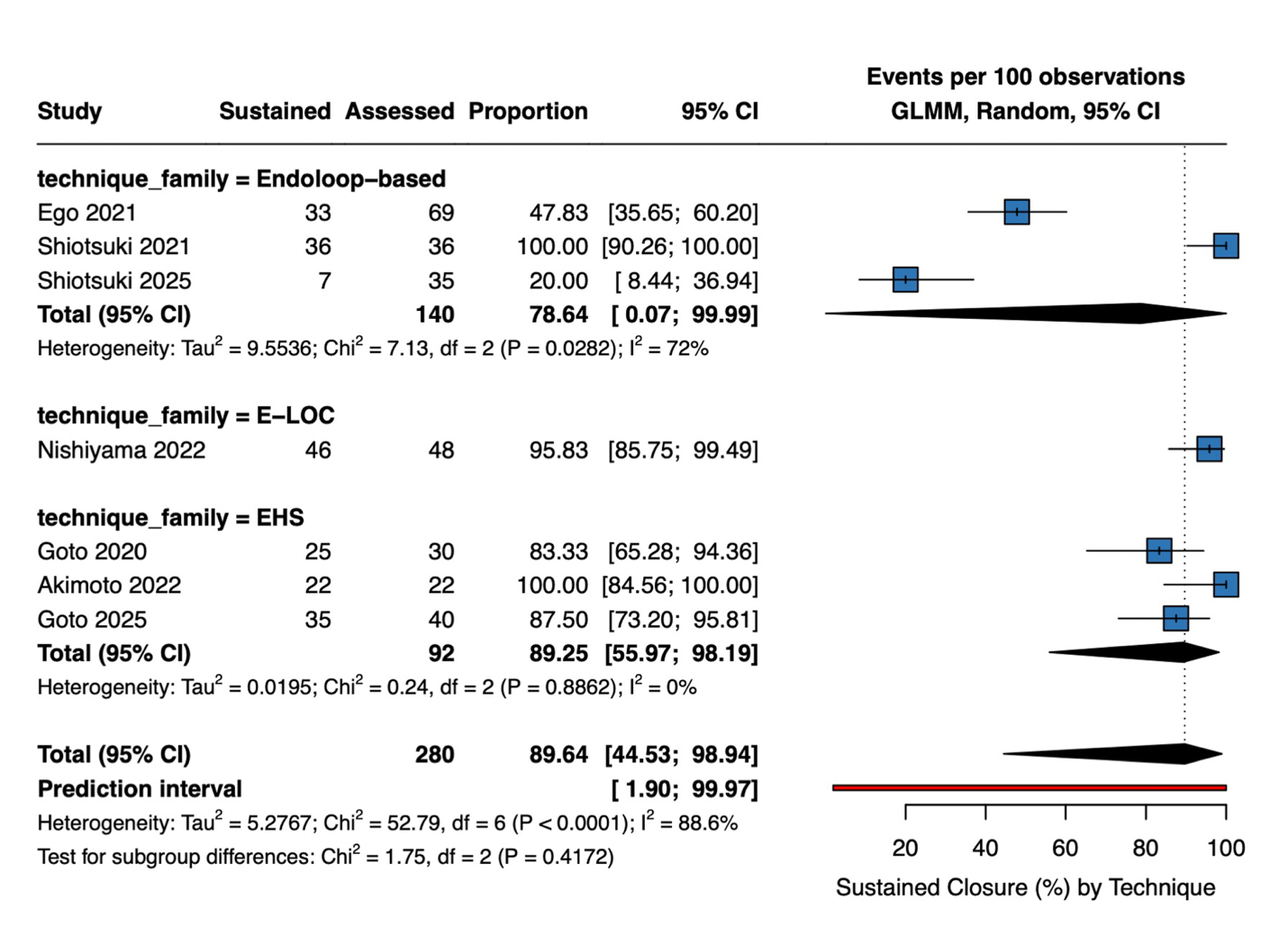
**

Panel B: Subgroup forest plot stratifying 7 cohorts by technique family. Endoloop-based (Ego 2021, Shiotsuki 2021, Shiotsuki 2025): 78.6% (95% CI 0.07-99.99%), I²=72%, n=140, marked heterogeneity driven by assessment timing variation. Endoscopic ligation with O-ring closure (Nishiyama 2022 only): 95.8% (95% CI 85.8-99.5%), n=48, highest sustained rate with early assessment (postoperative day 2-3). Endoscopic hand suturing (Goto 2020, Akimoto 2022, Goto 2025): 89.3% (95% CI 55.97-98.19%), I²=0%, n=92, homogeneous across three independent cohorts. Test for subgroup difference: χ²=1.75, p=0.42, not statistically significant.

**Supplementary Figure S9. Closure Procedure Time - Expanded Analysis**

**
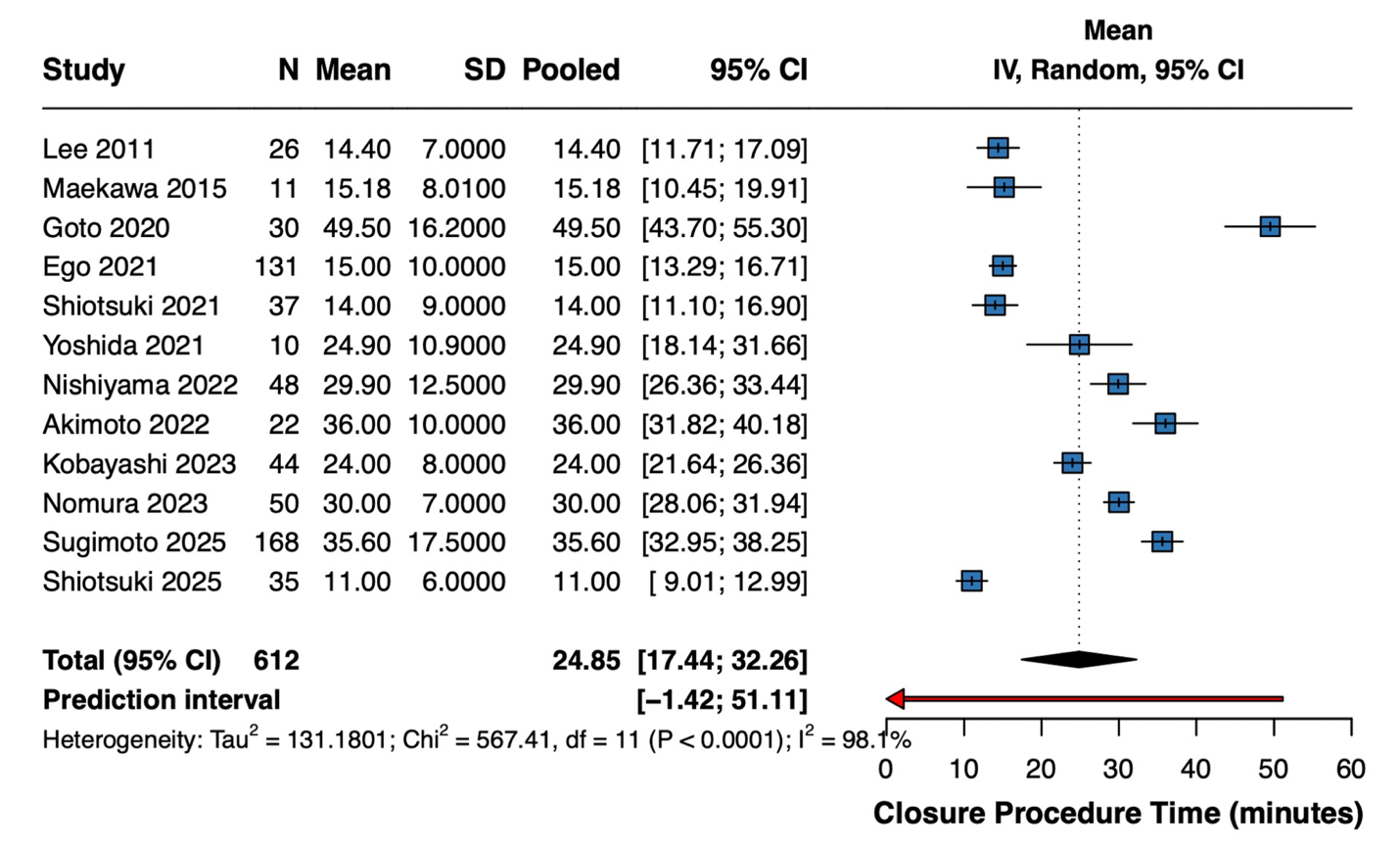
**

Panel A: Forest plot displaying weighted mean closure procedure times from 12 studies with extractable time data (n=612 procedures). Random-effects model for continuous outcomes using inverse-variance weighting with Hartung-Knapp adjustment. Pooled mean 24.9 minutes (95% CI 17.4-32.3), I²=98.1%. Five studies reported mean±SD directly; seven provided median with range/IQR requiring Wan/Luo method transformation. Individual mean times ranged from 11.0 minutes (Shiotsuki 2025 FLEXLOOP) to 49.5 minutes (Goto 2020 endoscopic hand suturing). Goto 2025 provided mean 48 minutes without SD, excluded from pooling.

**
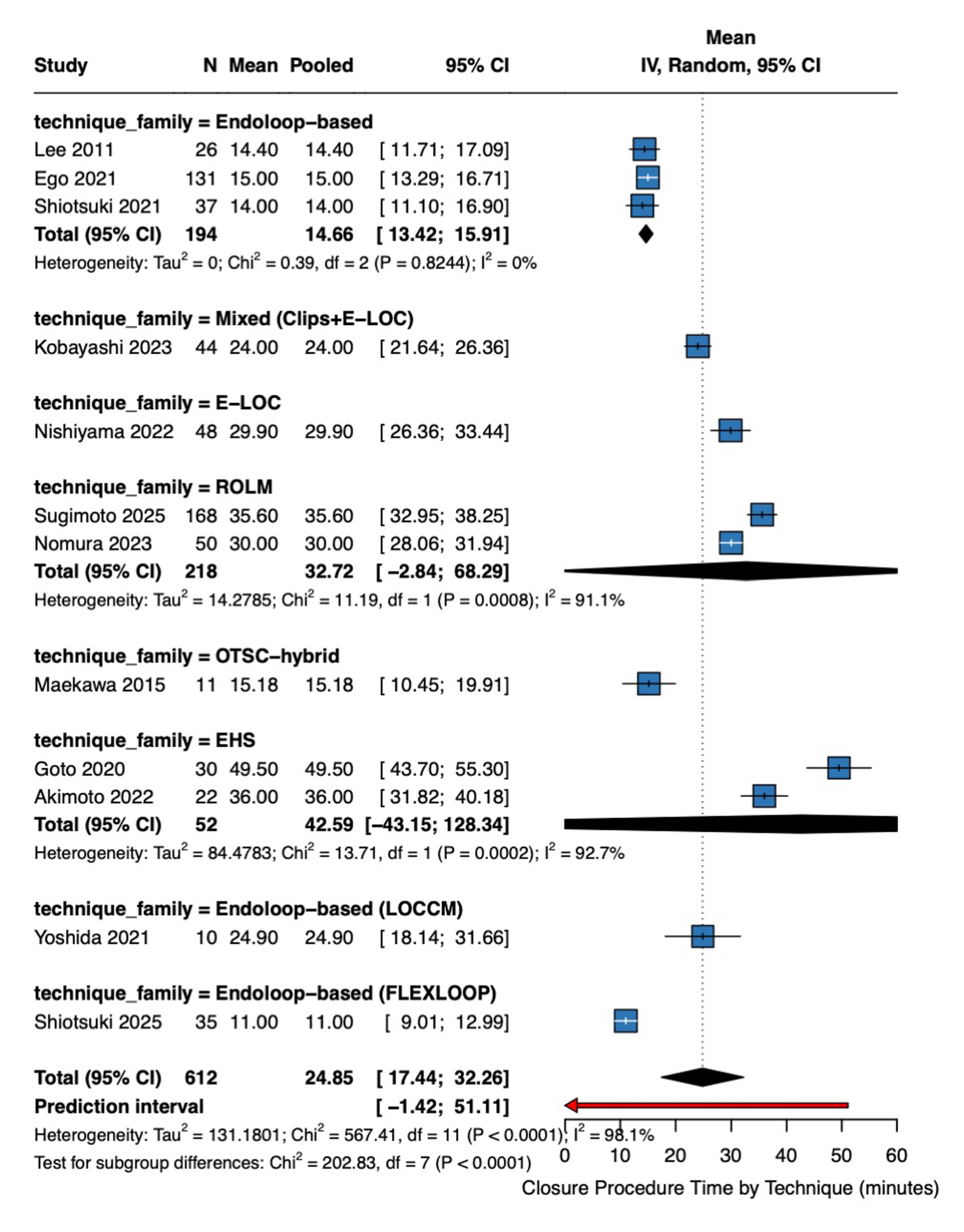
**

Panel B: Subgroup forest plot stratifying 12 studies by technique family (8 categories). Endoloop-based (Lee 2011, Ego 2021, Shiotsuki 2021): mean 14.7 minutes (95% CI 13.4-15.9), I²=0%. Mixed Clips+E-LOC (Kobayashi 2023): 24.0 minutes. E-LOC (Nishiyama 2022): 29.9 minutes. ROLM (Sugimoto 2025, Nomura 2023): 32.7 minutes (95% CI -2.8 to 68.3), I²=91.1%. OTSC-hybrid (Maekawa 2015): 15.2 minutes. EHS (Goto 2020, Akimoto 2022): 42.6 minutes (95% CI -43.2 to 128.3), I²=92.7%. LOCCM (Yoshida 2021): 24.9 minutes. FLEXLOOP (Shiotsuki 2025): 11.0 minutes. Test for subgroup differences: χ²=202.83, df=7, p<0.0001.

**Supplementary Figure S10. Closure-Related Adverse Events - Expanded Analysis**

**
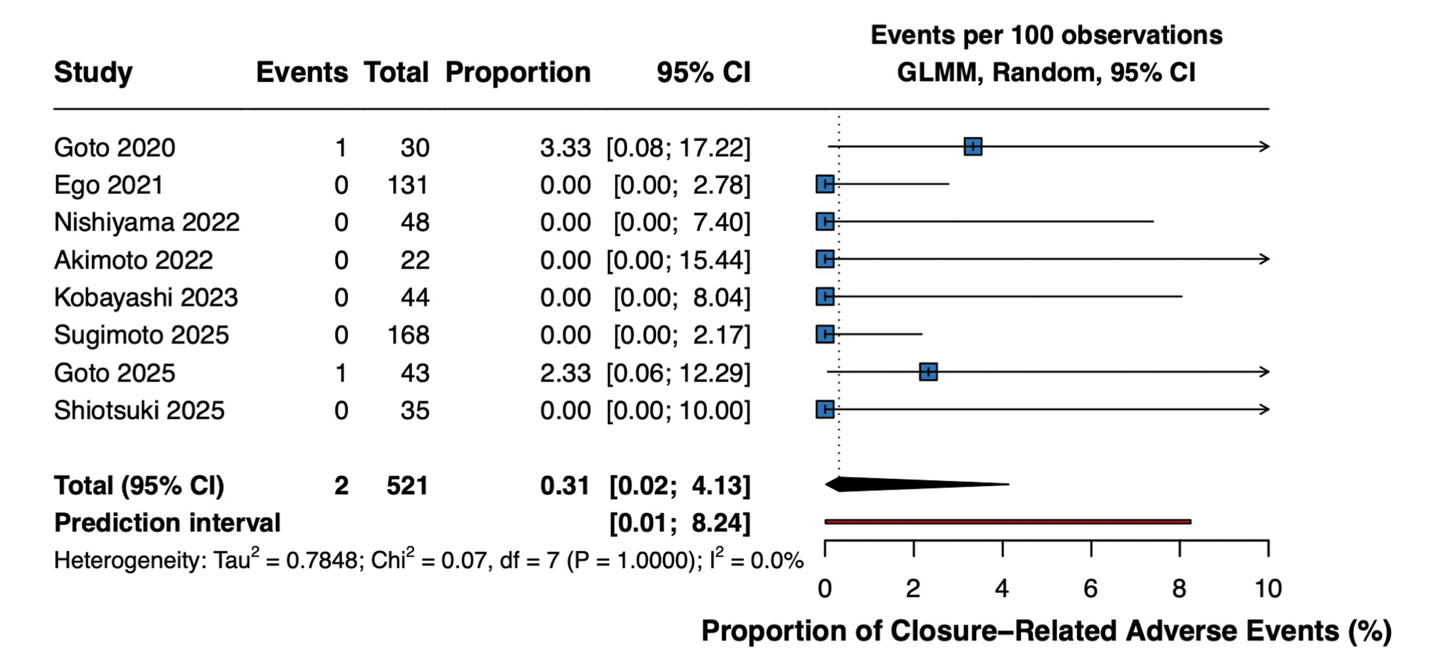
**

Forest plot of single-arm proportion meta-analysis for closure-related adverse events from 8 reporting closure cohorts (n=521 procedures: 4 comparative study closure arms, 4 single-arm studies). Random-effects model using generalized linear mixed model with logit link for rare event meta-analysis. Pooled proportion 0.31% (95% CI 0.02-4.13%), I²=0%, τ²=0.78, prediction interval 0.01-8.24%. Only 2 events total: Goto 2020 (1/30, needle puncture bleeding during endoscopic hand suturing) and Goto 2025 (1/43, minor mucosal erosion). Nine additional studies excluded as "not reported" because they did not explicitly assess closure-specific adverse events separately from general post-endoscopic submucosal dissection complications.

**Supplementary Figure S11. Funnel Plot for Publication Bias Assessment**


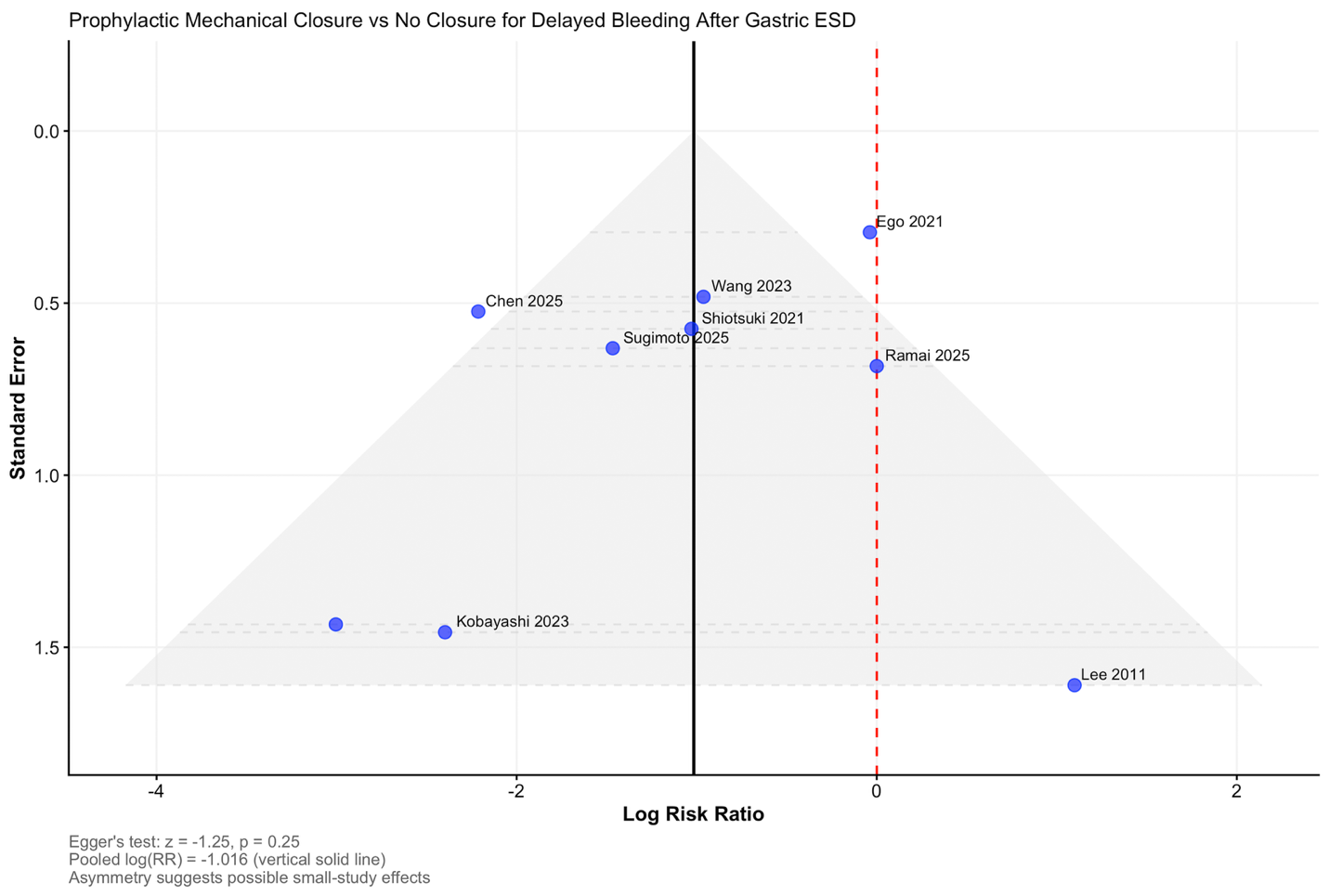


Funnel plot of nine comparative studies plotting log risk ratio for clinically significant delayed bleeding (x-axis) against standard error (y-axis). Vertical line indicates pooled effect under random-effects model: log RR=-1.02 (equivalent to RR 0.36). Egger's regression test for asymmetry: intercept=-3.42, z=-1.25, p=0.25 (not statistically significant). Visual inspection suggests possible asymmetry with apparent underrepresentation of small studies showing null or harmful effects; however, statistical test lacks power with only 9 studies (reliable Egger's test typically requires ≥10 studies).
