# Supplementary material for: Prophylactic Mechanical Closure for Preventing Delayed Bleeding after Gastric Endoscopic Submucosal Dissection: A Systematic Review and Meta‐Analysis
Source: DEN Open. 2026 Feb 14;6(1):e70299. doi: 10.1002/deo2.70299 (PMC12906296; doi:10.1002/deo2.70299)
Supplement: Supplementary file 2 — Appendix S1: Detailed Search Strategy Comprehensive five‐database systematic search strategy was conducted in October 2025. Total 476 citations identified. [file DEO2-6-e70299-s003.docx]

**SUPPLEMENTAL APPENDIX S1: DETAILED SEARCH STRATEGY**

**Comprehensive Five-Database Systematic Search**

A comprehensive systematic literature search was conducted across five electronic databases and one clinical trial registry on October 01, 2025, following PRISMA 2020 guidelines and Cochrane Handbook recommendations. The search strategy was developed in collaboration with a medical librarian and validated by identifying landmark studies known to meet our inclusion criteria. No language or date restrictions were applied to maximize search sensitivity. The complete search strategies for each database are detailed below.

**1. PubMed/MEDLINE**

**Platform:** https://pubmed.ncbi.nlm.nih.gov/
**Interface:** PubMed.gov Advanced Search
**Coverage:** 1946 to present

**Search Strategy:**

(("Stomach"[Mesh] OR "gastric"[Title/Abstract] OR "stomach"[Title/Abstract])

AND ("Endoscopic Mucosal Resection"[Mesh] OR "endoscopic submucosal

dissection"[Title/Abstract] OR "ESD"[Title/Abstract] OR "endoscopic

resection"[Title/Abstract]) AND ("Surgical Procedures, Operative"[Mesh:NoExp]

OR "closure"[Title/Abstract] OR "suturing"[Title/Abstract] OR

"clips"[Title/Abstract] OR "clipping"[Title/Abstract] OR "endoscopic hand

suturing"[Title/Abstract] OR "EHS"[Title/Abstract] OR "ROLM"[Title/Abstract]

OR "reopenable clip"[Title/Abstract] OR "over-the-scope clip"[Title/Abstract]

OR "OTSC"[Title/Abstract] OR "endoloop"[Title/Abstract] OR "prophylactic

closure"[Title/Abstract] OR "defect closure"[Title/Abstract] OR "mucosal

closure"[Title/Abstract]) AND ("Hemorrhage"[Mesh] OR

"bleeding"[Title/Abstract] OR "delayed bleeding"[Title/Abstract] OR

"post-procedural bleeding"[Title/Abstract] OR "post-ESD

bleeding"[Title/Abstract] OR "postprocedural bleeding"[Title/Abstract] OR

"hemorrhage"[Title/Abstract] OR "haemorrhage"[Title/Abstract]))

AND "humans"[MeSH Terms] NOT (animals[MeSH Terms] NOT humans[MeSH Terms])

**Results:** 105 citations
**Filters Applied:** Human studies only; no language restrictions; no date restrictions
**Rationale:** PubMed/MEDLINE was searched using a combination of MeSH terms and free-text keywords to capture both indexed and recently published articles. The strategy balanced sensitivity and specificity by combining controlled vocabulary (MeSH) with text words to maximize retrieval of relevant studies on prophylactic closure after gastric endoscopic submucosal dissection.

**2. Scopus**

**Platform:** https://www.scopus.com/
**Interface:** Scopus Advanced Search
**Coverage:** 1966 to present

**Search Strategy:**

TITLE-ABS-KEY((gastric OR stomach) AND ("endoscopic submucosal dissection"

OR "ESD" OR "endoscopic resection") AND (closure OR suturing OR clips OR

clipping OR "endoscopic hand suturing" OR "EHS" OR "ROLM" OR "reopenable

clip" OR "over-the-scope clip" OR "OTSC" OR endoloop OR "prophylactic

closure" OR "defect closure" OR "mucosal closure") AND (bleeding OR "delayed

bleeding" OR "post-procedural bleeding" OR "post-ESD bleeding" OR

"postprocedural bleeding" OR hemorrhage OR haemorrhage))

**Results:** 299 citations
**Filters Applied:** None (all document types, subject areas, and languages included)
**Document Types:** All types included (article, review, conference paper)
**Date Range:** All years (1966-present)
**Validation:** Search strategy successfully identified landmark studies including Sugimoto 2024, Ramai 2025, and Goto 2020, confirming adequate sensitivity
**Rationale:** Scopus provides broader coverage than PubMed, including conference proceedings, early online publications, and journals not indexed in MEDLINE. The title-abstract-keyword search ensures comprehensive retrieval across multiple document types.

**3. Embase (Classic + Embase)**

**Platform:** Embase.com (Elsevier)
**Interface:** Advanced Search
**Coverage:** 1947 to present

**Search Strategy:**

('stomach'/exp OR 'gastric':ab,ti OR 'stomach':ab,ti) AND ('endoscopic

mucosal resection'/exp OR 'endoscopic submucosal dissection':ab,ti OR

'esd':ab,ti) AND ('closure':ab,ti OR 'suturing':ab,ti OR 'clips':ab,ti OR

'clipping':ab,ti OR 'endoscopic hand suturing':ab,ti OR 'ehs':ab,ti OR

'rolm':ab,ti OR 'reopenable clip':ab,ti OR 'over-the-scope clip':ab,ti OR

'otsc':ab,ti OR 'prophylactic closure':ab,ti OR 'defect closure':ab,ti) AND

('bleeding':ab,ti OR 'delayed bleeding':ab,ti OR 'post-esd bleeding':ab,ti OR

'postprocedural bleeding':ab,ti OR 'hemorrhage':ab,ti)

AND 'human'/de AND [embase]/lim NOT [medline]/lim AND [article]/lim

**Results:** 36 citations
**Filters Applied:**

- Human studies only: 'human'/de
- Embase-unique records only (excluding Medline overlap): [embase]/lim NOT [medline]/lim
- Article type only: [article]/lim (excluding conference abstracts, editorials, letters)
- No language restrictions
- No date restrictions

**Rationale:** Embase search was strategically limited to Embase-unique records (excluding Medline/PubMed overlap) and article publications only to capture high-quality studies indexed in Embase but not available in PubMed. This approach avoids duplication while maximizing coverage of European journals, pharmaceutical literature, and early online publications. The restriction to article type excludes preliminary conference abstracts that typically lack peer review, focusing on definitive peer-reviewed publications.

**4. Cochrane Central Register of Controlled Trials (CENTRAL)**

**Platform:** Ovid (OvidSP)
**Interface:** Ovid EBM Reviews - Cochrane Central Register of Controlled Trials
**Coverage:** Current issue (all years available in CENTRAL)

**Search Strategy (Combined):**

((exp Stomach/ or (gastric or stomach).mp.) and (exp Endoscopic Mucosal

Resection/ or (endoscopic submucosal dissection or ESD or endoscopic

resection).mp.) and ((closure or suturing or clips or clipping or endoscopic

hand suturing or EHS or ROLM or reopenable clip or over-the-scope clip or

OTSC or endoloop or prophylactic closure or defect closure).mp.) and (exp

Hemorrhage/ or (bleeding or delayed bleeding or post-procedural bleeding or

post-ESD bleeding or postprocedural bleeding or hemorrhage or

haemorrhage).mp.))

**Line-by-Line Search Strategy:**

1. exp Stomach/ or (gastric or stomach).mp.
2. exp Endoscopic Mucosal Resection/ or (endoscopic submucosal dissection or ESD or endoscopic resection).mp.
3. 1 and 2
4. (closure or suturing or clips or clipping or endoscopic hand suturing or EHS or ROLM or reopenable clip or over-the-scope clip or OTSC or endoloop or prophylactic closure or defect closure or mucosal closure).mp.
5. exp Hemorrhage/ or (bleeding or delayed bleeding or post-procedural bleeding or post-ESD bleeding or postprocedural bleeding or hemorrhage or haemorrhage).mp.
6. 3 and 4 and 5 **[FINAL RESULTS]**

**Results:** 27 citations
**Limits Applied:** None (CENTRAL includes controlled trials by default)
**Language:** No restrictions
**Date Range:** All years available in CENTRAL database

**Note on Ovid Search Fields:** The .mp. field searches title, abstract, original title, name of substance word, subject heading word, floating sub-heading word, keyword heading word, organism supplementary concept word, protocol supplementary concept word, rare disease supplementary concept word, unique identifier, and synonyms for maximum search sensitivity.

**Rationale:** Cochrane CENTRAL search captures randomized controlled trials and controlled clinical trials including those from conference proceedings and trial registries that may not be fully indexed in MEDLINE or other databases. This ensures comprehensive identification of all available randomized controlled trial evidence on prophylactic closure after gastric endoscopic submucosal dissection, which is critical for evidence synthesis.

**5. ClinicalTrials.gov**

**Platform:** https://clinicaltrials.gov/
**URL:** https://classic.clinicaltrials.gov/ct2/search/advanced
**Interface:** Advanced Search (Expert Search Query)

**Search Strategy:**

(gastric OR stomach) AND ("endoscopic submucosal dissection" OR ESD) AND

(closure OR clipping OR clips OR suturing OR "endoscopic hand suturing" OR

ROLM OR "over-the-scope clip" OR OTSC OR endoloop OR "prophylactic closure")

AND (bleeding OR hemorrhage OR haemorrhage)

**Results:** 9 registered clinical trials identified

**Filters Applied:**

- Study Status: All statuses included (recruiting, completed, terminated, withdrawn, unknown status)
- Study Results: All studies included (with and without posted results)
- Study Type: All types included (interventional and observational)
- No date restrictions
- No geographic restrictions

**Rationale:** ClinicalTrials.gov search identifies registered trials including ongoing studies and completed trials that may not yet be published in peer-reviewed journals. This is essential for:

1. Identifying unpublished data to address potential publication bias
2. Contacting investigators for preliminary or unpublished results from completed trials
3. Documenting ongoing research for discussion section context
4. Ensuring comprehensive evidence synthesis per PRISMA 2020 recommendations

**Action Items:**

- Extracted data from trials with posted results on ClinicalTrials.gov
- Contacted principal investigators of completed trials without posted results for additional data
- Monitored ongoing trials for completion and results posting during review process

**Search Results Summary**

**Total Citations Retrieved:** 476 citations across all five databases

- PubMed/MEDLINE: 105 citations
- Scopus: 299 citations
- Embase (unique records): 36 citations
- Cochrane CENTRAL: 27 citations
- ClinicalTrials.gov: 9 registered trials

**Deduplication:** 129 duplicate records removed using EndNote reference management software (version 21), leaving 347 unique citations for screening

**Records identified through database searching**

n = 476

**Duplicate records removed**

n = 129

**Records screened (title/abstract)**

n = 347

**Records excluded (title/abstract)**

n = 287

**Full-text articles assessed for eligibility**

n = 60

**Full-text articles excluded, with reasons**

n = 43

• Wrong intervention (n = 16)

• Wrong population (n = 11)

• Review articles (n = 6)

• Wrong study design (n = 8)

• Other reasons (n = 2)

**Studies included in qualitative synthesis**

n = 17

• Comparative studies (n = 9): 2,646 patients

• Single-arm studies (n = 8): Supplementary

**Studies included in quantitative synthesis**

**• Primary efficacy analysis: 9 comparative studies**

**• Technical outcomes: 17 total studiesStudies Included in Review:**

- **Comparative studies (primary analysis):** 9 studies
  - Ramai 2025
  - Chen 2025
  - Sugimoto 2025
  - Kobayashi 2023
  - Ego 2021
  - Shiotsuki 2021
  - Lee 2011
  - Wang 2023 (Chinese language, translated)
  - Nishiyama 2022 (historical control design)
- **Single-arm studies (supplementary technical feasibility analysis):** 8 studies
  - Maekawa 2015
  - Goto 2020
  - Shiotsuki 2025
  - Yoshida 2021
  - Kinoshita 2020
  - Nomura 2023
  - Akimoto 2022
  - Goto 2025

**Search Strategy Development and Validation**

The search strategy was developed iteratively with the following considerations:

**1. Concept Mapping:**

- **Population:** Gastric/stomach AND endoscopic submucosal dissection
- **Intervention:** Prophylactic closure techniques (clips, suturing, endoloop methods)
- **Comparator:** No closure or alternative closure techniques
- **Outcome:** Delayed bleeding/hemorrhage

**2. Search Term Selection:** Search terms were identified through:

- MeSH (Medical Subject Headings) and Emtree thesaurus review
- Text word analysis of key articles identified a priori
- Consultation with content experts in interventional endoscopy
- Review of terminology variations across geographic regions (e.g., "haemorrhage" vs "hemorrhage")

**3. Strategy Validation:** Search strategies were validated by ensuring retrieval of known landmark studies:

- Sugimoto 2024 (ROLM multicenter study)
- Ramai 2025 (recent large cohort)
- Goto 2020 (endoscopic hand suturing pilot)
- Lee 2011 (early clip closure study)

**4. Sensitivity vs Specificity Balance:** The search strategy prioritized sensitivity over specificity to minimize risk of missing relevant studies. High sensitivity was achieved through:

- Broad MeSH term explosion (e.g., exp Hemorrhage/)
- Comprehensive text word synonyms and acronyms
- Inclusion of both British and American spelling variants
- No language or date restrictions

The resulting number needed to screen (NNS = 347/17 = 20.4) represents an acceptable balance between sensitivity and screening burden for this clinically focused systematic review.

**Grey Literature and Additional Sources**

Beyond electronic database searching, the following additional sources were searched:

**1. Reference List Screening:**

- Manually reviewed reference lists of all included studies
- Reviewed reference lists of relevant systematic reviews and meta-analyses
- No additional studies meeting inclusion criteria identified through this method

**2. Citation Tracking:**

- Forward citation searching performed using Google Scholar and Scopus
- Identified subsequent studies citing included articles
- No additional eligible studies identified

**3. Clinical Trial Registries:**

- ClinicalTrials.gov searched as primary registry (9 trials identified)
- WHO International Clinical Trials Registry Platform (ICTRP) reviewed for non-US trials
- Japanese Clinical Trials Registry (jRCT) consulted given predominance of Japanese research in this field

**4. Conference Proceedings:**

- Major gastroenterology conference abstracts captured through Scopus and Embase searches
- Conference abstracts excluded if insufficient data for quality assessment or if subsequently published as full articles

**Search Updates and Study Selection Process**

**Search Currency:**

- Initial search completed: October, 2025

**Study Selection Process:** All citations were imported into Rayyan systematic review software (https://www.rayyan.ai/) for title/abstract screening. Two independent reviewers (initials blinded) screened all titles and abstracts against predetermined inclusion criteria. Conflicts were resolved through discussion or consultation with a third reviewer when necessary. Full-text articles were retrieved for all potentially eligible studies identified during title/abstract screening.

**Inter-rater Reliability:**

- Cohen's kappa for title/abstract screening: κ = 0.78 (substantial agreement, 94.2% observed agreement)
- Cohen's kappa for full-text screening: κ = 0.85 (almost perfect agreement, 96.3% observed agreement)
- High inter-rater agreement confirmed appropriate application of inclusion/exclusion criteria

**Language Considerations:**

- One Chinese-language article (Wang 2023) identified and included after professional translation
- One Japanese-language article screened but excluded (wrong study design)
- All other included studies were published in English

**Search Reproducibility**

Complete search strategies with date stamps are archived and available upon request to ensure full transparency and reproducibility. The search strategies documented here allow for:

1. Independent verification of search comprehensiveness
2. Replication of the search for future updates
3. Assessment of search quality by peer reviewers and readers
4. Development of search alerts for ongoing surveillance

All search strategies, screening decisions, and data extraction forms have been retained and are available as supplementary materials or upon request from the corresponding author.

**Search Conducted by:** Hariruk Yodying
**Search Strategy Peer-Reviewed by:** Patcharaon Petchkaewkul
**PROSPERO Registration:** [CRD420251172925]
