# Supplementary material for: Prophylactic Mechanical Closure for Preventing Delayed Bleeding after Gastric Endoscopic Submucosal Dissection: A Systematic Review and Meta‐Analysis
Source: DEN Open. 2026 Feb 14;6(1):e70299. doi: 10.1002/deo2.70299 (PMC12906296; doi:10.1002/deo2.70299)
Supplement: Supplementary file 3 — Completed PRISMA 2020 checklist for systematic reviews, indicating the location of each checklist item within the manuscript and supplementary materials. Complete supplementary materials are available online, including PRISMA 2020 checklist, detailed search strategies (Appendix S1), characteristics of additional single‐arm studies (Table S1), risk of bias assessment (Table S2), sensitivity analyses (Figures S1–S5), length of hospital stay (Figure S6), expanded technical feasibility outcomes (Figures S7–S10), and publication bias assessment (Figure S11). [file DEO2-6-e70299-s001.docx]

**PRISMA 2020 Checklist**

**Prophylactic Mechanical Closure for Preventing Delayed Bleeding After Gastric Endoscopic Submucosal Dissection: A Systematic Review and Meta-Analysis**

| **Section and Topic** | **Item #** | **Checklist Item** | **Location** |
| --- | --- | --- | --- |
| **TITLE** |  |  |  |
| Title | 1 | Identify the report as a systematic review. | Title page |
| **ABSTRACT** |  |  |  |
| Abstract | 2 | See the PRISMA 2020 for Abstracts checklist. | Abstract (222 words) |
| **INTRODUCTION** |  |  |  |
| Rationale | 3 | Describe the rationale for the review in the context of existing knowledge. | Introduction, para 1-3 |
| Objectives | 4 | Provide an explicit statement of the objective(s) or question(s) the review addresses. | Introduction, final para |
| **METHODS** |  |  |  |
| Eligibility criteria | 5 | Specify the inclusion and exclusion criteria for the review and how studies were grouped for the syntheses. | Methods - Study Selection; Table 1 |
| Information sources | 6 | Specify all databases, registers, websites, organisations, reference lists and other sources searched or consulted to identify studies. Specify the date when each source was last searched or consulted. | Methods - Search Strategy; Appendix S1 |
| Search strategy | 7 | Present the full search strategies for all databases, registers and websites, including any filters and limits used. | Appendix S1 |
| Selection process | 8 | Specify the methods used to decide whether a study met the inclusion criteria of the review, including how many reviewers screened each record and each report retrieved, whether they worked independently, and if applicable, details of automation tools used in the process. | Methods - Study Selection |
| Data collection process | 9 | Specify the methods used to collect data from reports, including how many reviewers collected data from each report, whether they worked independently, any processes for obtaining or confirming data from study investigators, and if applicable, details of automation tools used in the process. | Methods - Data Extraction |
| Data items | 10a | List and define all outcomes for which data were sought. Specify whether all results that were compatible with each outcome domain in each study were sought (e.g. for all measures, time points, analyses), and if not, the methods used to decide which results to collect. | Methods - Outcomes |
|  | 10b | List and define all other variables for which data were sought (e.g. participant and intervention characteristics, funding sources). Describe any assumptions made about any missing or unclear information. | Methods - Data Extraction; Table 1 |
| Study risk of bias assessment | 11 | Specify the methods used to assess risk of bias in the included studies, including details of the tool(s) used, how many reviewers assessed each study and whether they worked independently, and if applicable, details of automation tools used in the process. | Methods - Risk of Bias Assessment |
| Effect measures | 12 | Specify for each outcome the effect measure(s) (e.g. risk ratio, mean difference) used in the synthesis or presentation of results. | Methods - Statistical Analysis |
| Synthesis methods | 13a | Describe the processes used to decide which studies were eligible for each synthesis (e.g. tabulating the study intervention characteristics and comparing against the planned groups for each synthesis (item #5)). | Methods - Statistical Analysis |
|  | 13b | Describe any methods required to prepare the data for presentation or synthesis, such as handling of missing summary statistics, or data conversions. | Methods - Statistical Analysis |
|  | 13c | Describe any methods used to tabulate or visually display results of individual studies and syntheses. | Methods - Statistical Analysis |
|  | 13d | Describe any methods used to synthesize results and provide a rationale for the choice(s). If meta-analysis was performed, describe the model(s), method(s) to identify the presence and extent of statistical heterogeneity, and software package(s) used. | Methods - Statistical Analysis |
|  | 13e | Describe any methods used to explore possible causes of heterogeneity among study results (e.g. subgroup analysis, meta-regression). | Methods - Subgroup and Sensitivity Analyses |
|  | 13f | Describe any sensitivity analyses conducted to assess robustness of the synthesized results. | Methods - Subgroup and Sensitivity Analyses |
| Reporting bias assessment | 14 | Describe any methods used to assess risk of bias due to missing results in a synthesis (arising from reporting biases). | Methods - Publication Bias |
| Certainty assessment | 15 | Describe any methods used to assess certainty (or confidence) in the body of evidence for an outcome. | Methods - GRADE Assessment |
| **RESULTS** |  |  |  |
| Study selection | 16a | Describe the results of the search and selection process, from the number of records identified in the search to the number of studies included in the review, ideally using a flow diagram. | Results - Study Selection; Figure 1 |
|  | 16b | Cite studies that might appear to meet the inclusion criteria, but which were excluded, and explain why they were excluded. | Results - Study Selection; Figure 1 |
| Study characteristics | 17 | Cite each included study and present its characteristics. | Results - Study Characteristics; Table 1 |
| Risk of bias in studies | 18 | Present assessments of risk of bias for each included study. | Results - Risk of Bias; Table S2 |
| Results of individual studies | 19 | For all outcomes, present, for each study: (a) summary statistics for each group (where appropriate) and (b) an effect estimate and its precision (e.g. confidence/credible interval), ideally using structured tables or plots. | Results; Figures 2-8; Forest plots |
| Results of syntheses | 20a | For each synthesis, briefly summarise the characteristics and risk of bias among contributing studies. | Results - Primary Outcome |
|  | 20b | Present results of all statistical syntheses conducted. If meta-analysis was done, present for each the summary estimate and its precision (e.g. confidence/credible interval) and measures of statistical heterogeneity. If comparing groups, describe the direction of the effect. | Results - all outcome sections; Figures 2-8 |
|  | 20c | Present results of all investigations of possible causes of heterogeneity among study results. | Results - Subgroup Analyses; Figures 3-5 |
|  | 20d | Present results of all sensitivity analyses conducted to assess the robustness of the synthesized results. | Results - Sensitivity Analyses; Table 2; Figures S1-S5 |
| Reporting biases | 21 | Present assessments of risk of bias due to missing results (arising from reporting biases) for each synthesis assessed. | Results - Publication Bias; Figure S11 |
| Certainty of evidence | 22 | Present assessments of certainty (or confidence) in the body of evidence for each outcome assessed. | Results - GRADE Assessment; Table 3 |
| **DISCUSSION** |  |  |  |
| Discussion | 23a | Provide a general interpretation of the results in the context of other evidence. | Discussion, para 1-4 |
|  | 23b | Discuss any limitations of the evidence included in the review. | Discussion - Limitations |
|  | 23c | Discuss any limitations of the review processes used. | Discussion - Limitations |
|  | 23d | Discuss implications of the results for practice, policy, and future research. | Discussion - Clinical Implications; Future Research; Conclusions |
| **OTHER INFORMATION** |  |  |  |
| Registration and protocol | 24a | Provide registration information for the review, including register name and registration number, or state that the review was not registered. | Methods; Data Availability (PROSPERO: CRD420251172925) |
|  | 24b | Indicate where the review protocol can be accessed, or state that a protocol was not prepared. | Data Availability |
|  | 24c | Describe and explain any amendments to information provided at registration or in the protocol. | N/A - No amendments |
| Support | 25 | Describe sources of financial or non-financial support for the review, and the role of the funders or sponsors in the review. | Funding |
| Competing interests | 26 | Declare any competing interests of review authors. | Disclosures |
| Availability of data, code and other materials | 27 | Report which of the following are publicly available and where they can be found: template data collection forms; data extracted from included studies; data used for all analyses; analytic code; any other materials used in the review. | Data Availability; Supplementary Materials |

**Figure and Table Reference Summary**

| **Item** | **Figure/Table** | **Content** |
| --- | --- | --- |
| 16a | Figure 1 | PRISMA Flow Diagram (476 citations → 9 comparative studies) |
| 17 | Table 1 | Characteristics of 9 comparative studies |
| 18 | Table S2 | Risk of bias assessment (RoB 2, ROBINS-I) |
| 19 | Figures 2-8 | Forest plots for all outcomes |
| 20b | Figure 2 | Primary outcome: Delayed bleeding (RR 0.36, 95% CI 0.16-0.82) |
| 20c | Figure 3 | Subgroup: Antithrombotic therapy status |
| 20c | Figure 4 | Subgroup: Defect size |
| 20c | Figure 5 | Subgroup: Closure technique |
| 20b | Figure 6 | Immediate complete closure rate |
| 20b | Figure 7 | Sustained closure at second-look |
| 20b | Figure 8 | Closure procedure time |
| 20d | Table 2 | Sensitivity analyses summary |
| 20d | Figures S1-S5 | Sensitivity analysis forest plots |
| 21 | Figure S11 | Funnel plot (publication bias) |
| 22 | Table 3 | GRADE evidence certainty assessment |
| 17 | Table S1 | Characteristics of 8 single-arm studies |

**Supplementary Materials Index**

| **Supplement** | **Description** |
| --- | --- |
| Appendix S1 | Detailed search strategies for all databases |
| Table S1 | Characteristics of 8 additional single-arm studies |
| Table S2 | Risk of bias assessment summary |
| Figure S1 | Sensitivity: High-quality studies only (RCT + PSM) |
| Figure S2 | Sensitivity: Excluding historical control |
| Figure S3 | Sensitivity: Strict bleeding definition |
| Figure S4 | Sensitivity: Excluding non-protocolized techniques |
| Figure S5 | Sensitivity: Alternative AT categorization |
| Figure S6 | Length of hospital stay (descriptive) |
| Figure S7 | Immediate complete closure - expanded (17 studies) |
| Figure S8 | Sustained closure - expanded analysis |
| Figure S9 | Closure procedure time - expanded |
| Figure S10 | Closure-related adverse events |
| Figure S11 | Funnel plot - publication bias assessment |

*From:* Page MJ, McKenzie JE, Bossuyt PM, Boutron I, Hoffmann TC, Mulrow CD, et al. The PRISMA 2020 statement: an updated guideline for reporting systematic reviews. BMJ 2021;372:n71. doi: 10.1136/bmj.n71.

This work is licensed under CC BY 4.0. To view a copy of this license, visit https://creativecommons.org/licenses/by/4.0/
